# Genomic Analysis of Rwandan G9P[8] Rotavirus Strains Pre- and Post-RotaTeq^®^ Vaccine Reveals Significant Distinct Sub-Clustering in a Post-Vaccination Cohort

**DOI:** 10.3390/v15122321

**Published:** 2023-11-25

**Authors:** Robyn-Lee Potgieter, Peter N. Mwangi, Milton T. Mogotsi, Jeannine Uwimana, Leon Mutesa, Narcisse Muganga, Didier Murenzi, Lisine Tusiyenge, Mapaseka L. Seheri, A. Duncan Steele, Jason M. Mwenda, Martin M. Nyaga

**Affiliations:** 1 Next Generation Sequencing Unit and Division of Virology, Faculty of Health Sciences, University of the Free State, Bloemfontein 9300, South Africa; potgieterr@ufs.ac.za (R.-L.P.); mwangipn@ufs.ac.za (P.N.M.); mogotsimt@ufs.ac.za (M.T.M.); 2 Department of Pediatrics, Kigali University Teaching Hospital, College of Medicine and Health Sciences, University of Rwanda, Kigali P.O. Box 4285, Rwanda; jeannineuw@gmail.com (J.U.); lmutesa@gmail.com (L.M.); nmuganga@gmail.com (N.M.); murenzididier77@gmail.com (D.M.); tuyislisine@gmail.com (L.T.); 3 Centre for Human Genetics, College of Medicine and Health Sciences, University of Rwanda, Kigali P.O. Box 4285, Rwanda; 4 Diarrheal Pathogens Research Unit, Sefako Makgatho Health Sciences University, Medunsa, Pretoria 0204, South Africa; mapaseka.seheri@smu.ac.za (M.L.S.); duncan.steele@gatesfoundation.org (A.D.S.); 5 World Health Organization, Regional Office for Africa, Brazzaville P.O. Box 06, Congo; mwendaj@who.int

**Keywords:** rotavirus, Rwanda, G9P[8], vaccination, whole-genomic analysis

## Abstract

Although the introduction of rotavirus vaccines has substantially contributed to the reduction in rotavirus morbidity and mortality, concerns persist about the re-emergence of variant strains that might alter vaccine effectiveness in the long term. The G9 strains re-emerged in Africa during the mid-1990s and have more recently become predominant in some countries, such as Ghana and Zambia. In Rwanda, during the 2011 to 2015 routine surveillance period, G9P[8] persisted during both the pre- and post-vaccine periods. The pre-vaccination cohort was based on the surveillance period of 2011 to 2012, and the post-vaccination cohort was based on the period of 2013 to 2015, excluding 2014. The RotaTeq^®^ vaccine that was first introduced in Rwanda in 2012 is genotypically heterologous to Viral Protein 7 (VP7) G9. This study elucidated the whole genome of Rwandan G9P[8] rotavirus strains pre- and post-RotaTeq^®^ vaccine introduction. Fecal samples from Rwandan children under the age of five years (pre-vaccine n = 23; post-vaccine n = 7), conventionally genotyped and identified as G9P[8], were included. Whole-genome sequencing was then performed using the Illumina^®^ MiSeq platform. Phylogenetic analysis and pair-wise sequence analysis were performed using MEGA6 software. Distinct clustering of three post-vaccination study strains was observed in all 11 gene segments, compared to the other Rwandan G9P[8] study strains. Specific amino acid differences were identified across the gene segments of these three 2015 post-vaccine strains. Important amino acid differences were identified at position N242S in the VP7 genome segment of the three post-vaccine G9 strains compared to the other G9 strains. This substitution occurs at a neutralization epitope site and may slightly affect protein interaction at that position. These findings indicate that the Rwandan G9P[8] strains revealed a distinct sub-clustering pattern among post-vaccination study strains circulating in Rwanda, with changes at neutralization epitopes, which may play a role in neutralization escape from vaccine candidates. This emphasizes the need for continuous whole-genome surveillance to better understand the evolution and epidemiology of the G9P[8] strains post-vaccination.

## 1. Introduction

Group A rotaviruses (RVAs) are a major viral etiological agent of acute and severe gastroenteritis among young children under the age of five years [1]. Extensive epidemiological data have demonstrated that by the age of five, an overwhelming 95% of children get infected by the virus, irrespective of their socioeconomic status [2]. Before the introduction of rotavirus vaccines, the average annual global mortality cases of rotavirus were estimated at 500,000 [2]. After the introduction of rotavirus vaccines, the mortality cases have significantly reduced to roughly 128,500 deaths with 81% of these deaths having occurred in developing countries in southeast Asia as well as sub-Saharan Africa [3]. This high rate of morbidity and mortality among RVA-related disease among children from low- and middle-income countries is due to challenges in accessing efficient medical care and living in settings with relatively poor facilities that cannot offer optimal rehydration services [3,4]. Other factors that may affect vaccine effectiveness include, among others, maternal antibodies, and high strain diversity of circulating strains in these regions compared to the developed world [5].

To combat the rotavirus disease burden, the World Health Organization (WHO) has prequalified four rotavirus vaccines for global use after extensive efficacy and safety studies [6]. In 2006, RotaTeq^®^ (Merck & Co. Inc., Kenilworth, NJ, USA) and Rotarix^®^ (GlaxoSmithKline Biologicals, Rixensart, Belgium) were the first two vaccines to be prequalified by the WHO [7]. Later in 2018, the WHO prequalified two additional vaccines: Rotavac^®^ (Bharat Biotech, Hyderabad, India) and Rotasiil^®^ (Serum Institute of India, Pune, India) (https://extranet.who.int/gavi/PQ_Web/ accessed on 17 March 2023). Rwanda introduced the RotaTeq^®^ vaccine in May 2012 and achieved 99% vaccination coverage within the first year of introduction [8]. This relatively high vaccination coverage resulted in a sharp decrease in diarrhea-associated hospitalization (25–44%) and hospitalization rates among Rwandan children in the first three years post-RotaTeq^®^ introduction [9]. However, in April 2017, Rwanda made a switch to the Rotarix^®^ vaccine due to economic considerations [9]. 

Rotavirus is classified in the *Sedoreoviridae* family and has a dsRNA genome of 18,552 bp (https://ictv.global/report/chapter/reovirales accessed on 26 February 2023). The RVA genome has 11 segments contained within icosahedral capsids [10]. The RVA gene translation produces 11 and sometimes 12 viral proteins, six structural viral proteins (VPs) identified as VP1-VP4, VP6 and VP7, and five or six non-structural proteins (NSPs) denoted as NSP1-5/6 [10,11]. Conventionally, a binary classification system derived from the immunological properties of the two outer capsid proteins (VP4 and VP7) was used and is still globally accepted to classify rotaviruses into G types and P types, respectively [10]. These proteins act as neutralization antigens, providing specificity to RVA strains [12]. With the increase of the use of next generation sequencing, the whole-genome classification scheme is rapidly becoming the more preferred way to classify rotaviruses. Based on this scheme, human RVAs were classified into three genogroups, the Wa-like and the DS-1-like, which are the two major genogroups, and the AU-1-like, which is a minor genogroup [10]. 

Globally, the most prevalent human RVA genotypes detected are G1P[8], G2P[4], G3P[8], G4P[8], G9P[8], and G12P[8] [13]. Together, they account for up to three quarters of human rotavirus infections worldwide [14]. However, in Africa, the most prevalent rotavirus genotype combinations detected between 2006 and 2008 were G1P[8], G2P[4], G9P[8], G12P[8], and G12P[6] [15]. The G9 strains re-emerged in Africa during the mid-1990s and have more recently become predominant in many countries [16]. The G9P[8] genotype was first identified in 1983 in the United States of America and quickly became prevalent globally [17]. The origin of the VP4 and VP7 genome segments for the vaccine strains are as follows: RotaTeq^®^—G1, G2, G3, G4, P[8], and G6P[5]; Rotarix^®^—G1P[8]; RotaVac^®^—G9P[11]; and Rotasiil^®^—G1, G2, G3, G4, G9, and G6P[5] [17,18].

The P[8] component is not present in some vaccine candidates such as the RotaVac^®^ and Rotasiil^®^ vaccine [18]. In Rwanda, the G1P[8] was the predominant strain circulating in 2011 (pre-vaccination era) [19]. In 2013 (post-vaccination era), G8P[4] become the dominant strain, accounting for 56% of cases [19]. The G2P[4] and G12P[6] genotypes became the predominant strains circulating in 2009 to 2010, with G1P[8] and G9P[8] circulating in 2011 to 2012 and the G9P[8] genotype dominating 2013, and in 2014 to 2015, the G12P[8] genotype was circulating in this region, showing how G9P[8] persisted during both the pre- and post-vaccine periods [19,20].

Although the rotavirus vaccines appear to be having a beneficial impact on the disease burden, there are concerns that their administration may be selecting for the emergence of potential vaccine-escape mutants [20,21]. Evolutionary mechanisms such as genome reassortment, and rearrangement, have resulted in the emergence of different novel RVA strains that may have the ability to circulate throughout the community [21]. These novel circulating strains may contribute to suboptimal effectiveness of the vaccines in regions such as the low-income countries in sub-Saharan Africa and southeast Asia, where strains are highly diverse [21]. Vaccines have shown higher effectiveness rates in high-income countries compared to low-income countries [22,23]. This emphasizes the importance of performing whole-genome analysis, which allows for a greater understanding of the changing aspects of RVA strains pre- and post-vaccination in Africa, specifically in low-income countries such as Rwanda [19,24,25]. Therefore, the purpose of this study was to assess any changes in the evolution of the genomic makeup of the G9P[8] strains that were circulating in Rwanda during the pre- and post-RotaTeq^®^ vaccination periods.

## 2. Materials and Methods

### 2.1. Ethical Statement

Ethical approval for this study was sought from the University of the Free State Health Sciences Research Ethics Committee (HSREC), where the study was granted approval under the reference UFS-HSD2022/0983/2709. The archived patient samples had all their personal information delinked and anonymized. 

### 2.2. Sample Description

Fecal samples (n = 158) from Rwandan children under the age of five years were sequenced at the University of the Free State Next Generation Sequencing Unit (UFS-NGS), Bloemfontein, South Africa. Of these samples, 46 were sequenced from the pre-vaccination era, and 112 from the post-vaccine era. Samples (n = 30) that had been previously conventionally genotyped for their VP7 and VP4 genes as G9P[8] were included in this study. From these successfully sequenced samples, 23 were from the pre-vaccination period and seven from post-vaccination period. The focus on the G9P[8] strains was due to their recognized epidemiological significance as well as being not only important in Rwanda, but also well recognized as a globally prevalent genotype. 

### 2.3. Rotavirus dsRNA Extraction and Purification

The total viral nucleic acid material was extracted as described previously [26]. Briefly, approximately 100 mg of stool sample was added to 200 µL of phosphate-buffered solution (PBS) (Sigma-Aldrich^®^, Saint Louis, MO, USA) to create a stool suspension. A total of 300 µL of the stool suspension was added to 900 µL of TRI Reagent^®^ (Sigma-Aldrich^®^, Saint Louis, MO, USA) after the stool suspension stood for 10 min at room temperature. Thereafter, the mixture was centrifuged (Eppendorf centrifuge 5427R, Hamburg, Germany) at 18,000 RPM for 20 min at 4 °C. A volume of 700 µL of ice-cold isopropanol (Sigma-Aldrich^®^, Saint Louis, MO, USA) was added, and the mixture was left to dry for 10 min to precipitate the supernatants. The extracted nucleic material was incubated in 8M LiCl2 (Sigma-Aldrich^®^, St Louis, MO, USA) at 4 °C for 16 h to enrich the dsRNA viruses and remove impurities. The extracted nucleic acid material was thereafter purified using a MinElute gel extraction kit (Qiagen, Hilden, Germany), and the integrity and enrichment of the dsRNA were verified via 1% agarose gel electrophoresis and visualized using an ultraviolet (UV) transilluminator (Sigma-Aldrich^®^, Saint Louis, MO, USA).

### 2.4. Double-Stranded cDNA Synthesis

Complementary DNA (cDNA) was synthesized on the purified enriched dsRNA by utilizing the optimized Maxima H Minus Double Stranded Synthesis Kit protocol (ThermoFisher Scientific, Waltham, MA, USA). This modified protocol entails a first- and second-strand cDNA synthesis step. Briefly, a 13 µL volume dsRNA was denatured for 5 min at 95 °C, allowing the dsRNA to unwind. The denatured RNA was then spun down for 10 s. A 1 µL volume of the random hexamer primer (ThermoFischer Scientific, Waltham, MA, USA) was added to 13 µL of the denatured dsRNA. The tubes were once again spun down and were then placed in a thermocycler (Labnet, Edison, NJ, USA) for 5 min to allow for annealing of the random hexamer primer to the template RNA. Thereafter, 5 µL of the 4 X first-strand reaction mix and 1 µL of the first-strand enzyme mix was added and they were incubated at 50 °C for 30 min. Second-strand synthesis proceeded immediately using the kit’s master mix, where 55 µL of nuclease-free water was added to the reaction mixture. This was followed by the addition of a 20 µL volume of 5× second-strand reaction mix, followed by the addition of 5 µL of second-strand enzyme mix (Thermo Fischer Scientific, Waltham, MA, USA). This mixture was then placed in the thermocycler (Labnet, Edison, NJ, USA) for 60 min at 16 °C to incubate. Thereafter, a 6 µL volume of 0.5 M EDTA (ThermoFisher Scientific, Waltham, MA, USA) with a pH of 8.0 was added to bring the reaction to a stop. A 10 µL volume RNase 1 (ThermoFisher Scientific, Waltham, MA, USA) was added to remove residual RNA, and this was further incubated for 5 min at room temperature. An MSB^®^ Spin PCRapace purification kit (Stratec Molecular, Berlin, Germany) was used to purify the synthesized cDNA. Purified cDNA was quantified using a Qubit™ 3.0 Fluorometer (Invitrogen, Carlsbad, CA, USA). 

### 2.5. Nextera^®^ XT DNA Library Preparation and Whole-Genome Sequencing

DNA libraries were prepared using the Nextera^®^ XT DNA Library Kit (Illumina, San Diego, CA, USA). Briefly, the template DNA was tagmented, followed by indexing, amplification, and clean-up of the genomic DNA using Ampure XP beads (Beckman Coulter, Pasadena, CA, USA). The quality of the libraries as well as the fragment size distribution were thereafter assessed using an Agilent 2100 Bioanalyzer (Agilent Technologies, Waldbronn, Germany). The libraries were normalized to 4 nM and pooled together for MiSeq^®^ sequencing. This was accomplished by combining 5 µL of the individually barcoded libraries into a single Eppendorf^®^ tube (Eppendorf AG, Hamburg, Germany). Thereafter, sodium hydroxide (NaOH) (Sigma-Aldrich^®^, Saint Louis, MO, USA) was used to denature the pooled libraries. The pooled libraries together with the NaOH resulted in a total volume of 10 µL, which was incubated to allow the DNA to denature into single strands. After denaturation occurred, 990 µL of pre-chilled hybridization buffer HT1 (Illumina, San Diego, CA, USA) was added, resulting in 20 pM denatured libraries. A further dilution of the 20 pM library was carried out to a sequencing concentration of 8 pM, and a denaturation step of the PhiX positive control (Illumina, San Diego, CA, USA) was added for optimal cluster density. A final volume of 600 µL of diluted library with a 2% PhiX control was loaded into a V3 (600 cycle) reagent kit (Illumina, San Diego, CA, USA), and whole-genome sequencing was performed at 301 × 2 paired-end reads using a MiSeq^®^ benchtop sequencer (Illumina, San Diego, CA, USA) at the UFS-NGS Unit, Bloemfontein, South Africa.

### 2.6. Data Analysis

Quality control of the raw data was performed using FASTQC v. 0.11.9 to proceed with a Phred data quality score of Q30 [27]. The Illumina sequence read ends were analyzed using Geneious Prime^®^ software v2022.0.1 (https://www.geneious.com, accessed on 26 February 2023; [28]), which comprised reference mapping to obtain full-length genomes. The study strains achieved 100% coverage of the ORF in the whole-genome sequencing, as well as 100% coverage of the genome, including the entire coding and noncoding regions. For phylogenetic analysis, the MUSCLE algorithm which is implemented in MEGA X (https://www.megasoftware.net/, accessed on 26 February 2023) was used to align the open reading frame (ORF) of each gene segment. After alignment, the DNA Model Test program in MEGA X was utilized for identification of the optimal evolutionary models for phylogenetic analysis. Thereafter, maximum likelihood trees were constructed together with approximately 30 random globally selected RVA reference strains for each gene segment with 1000-replicate bootstrap support, and the p-distance algorithm was used to calculate the genetic distance matrixes between amino acid and nucleotide sequences. The ORF sequences for all 11 genes of these Rwandan G9P[8] strains were sequenced and deposited in the NCBI GenBank under the references OR401005-OR401334.

## 3. Results

### 3.1. Whole-Genome Constellation Analysis

All 30 Rwandan G9P[8] strains from both the pre- and post-vaccination periods during the 2011 to 2015 surveillance season exhibited the typical Wa-like genotype constellation (G9-P[8]-I1-R1-C1-M1-A1-N1-T1-E1-H1). 

### 3.2. Comparative Analysis of the Neutralizing Epitope Regions in the VP7 and VP4 Genome Segments

Amino acid differences were observed when comparing the neutralization epitopes in the VP7 genome segment between the Rwandan G9P[8] study strains and the four WHO-prequalified vaccine strains. Within the antigenic epitope region of the VP7 genome segment [29], 26 of the 29 amino acids residues were conserved among the Rwandan G9 strains. The Rwandan study strains differed from some of the vaccine strains at three amino acid sites (96, 100, and 242) in the 7-1a and 7-1b antigenic epitope regions. The amino acid difference T96A, observed in one post-vaccination study strain, involved a polarity change from a polar to a nonpolar amino acid. This amino acid difference did not correlate with any of the vaccine candidates. When comparing the study strains to the vaccine strains, 20 of the Rwandan study strains (19 from the pre-vaccination era, and one from the post-vaccination era), including the RotaVac^®^ G9 vaccine strain, experienced the amino acid difference D100G, which involved a polarity change from a nonpolar to a polar amino acid. Three study strains from the post-vaccination era, with the inclusion of the RotaTeq^®^ G2 vaccine strain, demonstrated the amino acid difference N242S, which involved an amino acid substitution (Table 1). No amino acid differences were detected outside the VP7 epitopes. 

Antigenic analysis of the Rwandan G9P[8] study strains compared to the RotaTeq^®^, Rotarix^®^, Rotasiil^®^, and RotaVac^®^ vaccines was performed based on the three VP7 antigenic residues (7-1a, 7-1b, and 7-2). The amino acids highlighted in light green signify amino acid differences in the Rwandan study strains and the vaccine strains. The post-vaccine and pre-vaccine G9P[8] study strains are in bold and colored in red and black, respectively.

The neutralizing epitope regions of the VP4 genome segment comprise 37 amino acid residues [30]. Amino acid differences were observed when comparing the VP4 genome segment of the Rwandan G9P[8] study strains and the Rotarix^®^ and RotaTeq^®^ vaccines at three amino acid sites (195, 196, and 113) in the VP8* 8-1 and 8-3 antigenic epitope regions. The amino acid difference G195S, observed in one post-vaccination study strain, involved a change from polar to nonpolar. One Rwandan G9P[8] study strain from the pre-vaccine era, compared to the vaccine strains, demonstrated the amino acid difference I196L. Seven study strains, including three G9 study strains from the post-vaccination era, experienced the amino acid N113D (Table 2). When comparing the study strains to the RotaTeq^®^ vaccine, it is noted that the pre-vaccine study strains already differed from the vaccine strains at multiple epitopes, including one pre-vaccine strain (I96L) and four pre-vaccine strains (N113D). No amino acid differences were detected outside the VP4 epitopes.

Antigenic analysis of the 30 Rwandan G9P[8] study strains compared to the RotaTeq^®^ and Rotarix^®^ vaccines was carried out based on the two structural proteins (VP8* and VP5*) of the VP4 genome. The amino acids highlighted in light green and light yellow signify amino acid differences when comparing the Rwandan G9P[8] study strains and the vaccine strains. The post-vaccine and pre-vaccine G9P[8] study strains are in bold and colored in red and black, respectively.

### 3.3. Sequence and Phylogenetic Analysis of the VP7 and VP4 Genome Segments

**Figure 1:**
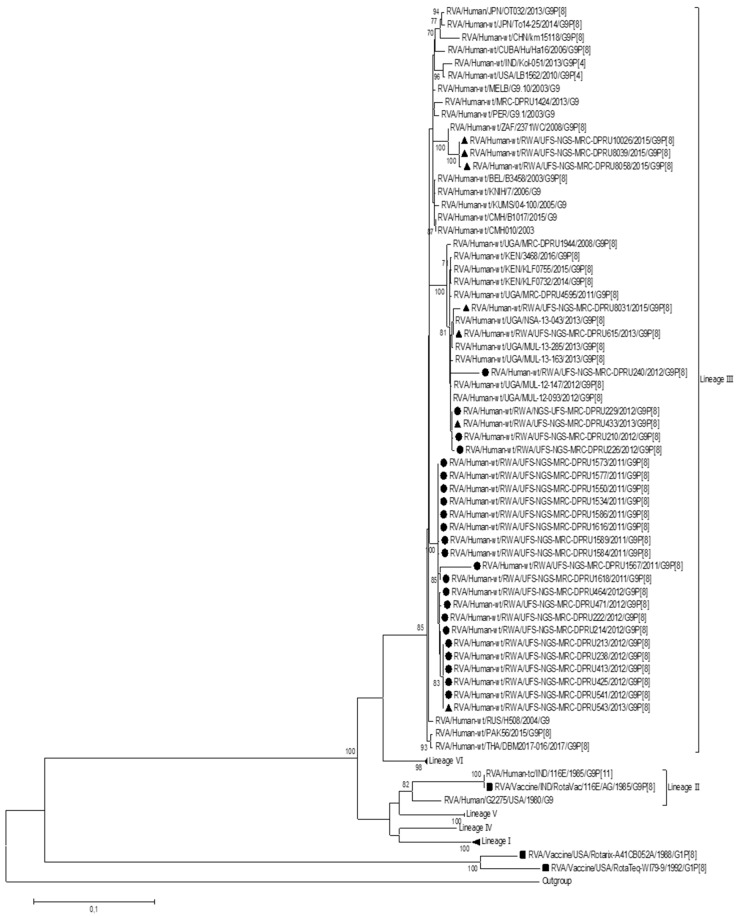
Maximum likelihood phylogenetic trees constructed from the nucleotide sequences of the Rwandan G9 strains for the VP7 genome. Using 1000 bootstrap replicates, the branch support was thoroughly evaluated. All G9P[8] study strains clustered into lineage III. The pre-vaccination study strains are indicated by a black circular shape, the post-vaccination study strains are indicated by a triangular shape, and the vaccine strains are indicated by a square shape.

In order to determine the genetic relationship between the Rwandan G9P[8] study strains and other global RVA strains, a phylogenetic analysis was performed. To construct the phylogenetic tree for the VP7 genome segment, lineage designation as per Gupta. Et al. was used, which defines six lineages for this genome segment (Figure 1). The Rwandan G9 study strains and global RVA reference G9 sequences for the VP7 genome segment were segregated into six lineages. All 30 of the Rwandan G9P[8] study strains clustered into lineage III, which comprised sequences predominantly from the Eastern African region. The Rwandan study strains within lineage III were closely related amongst each other, with nucleotide similarities ranging from 92.5% to 100%. Three post-vaccination study strains, which were identified to be noticeably sub-clustered together, demonstrated a nucleotide similarity range of 95,1% to 100%. Within lineage III, several sub-lineages were observed, where a 70% threshold node value was used to define a robust sub-lineage. Therefore, using this criterion for this study, we identified that Rwandan G9 sequences clustered into sub-lineage IIId. 

**Figure 2:**
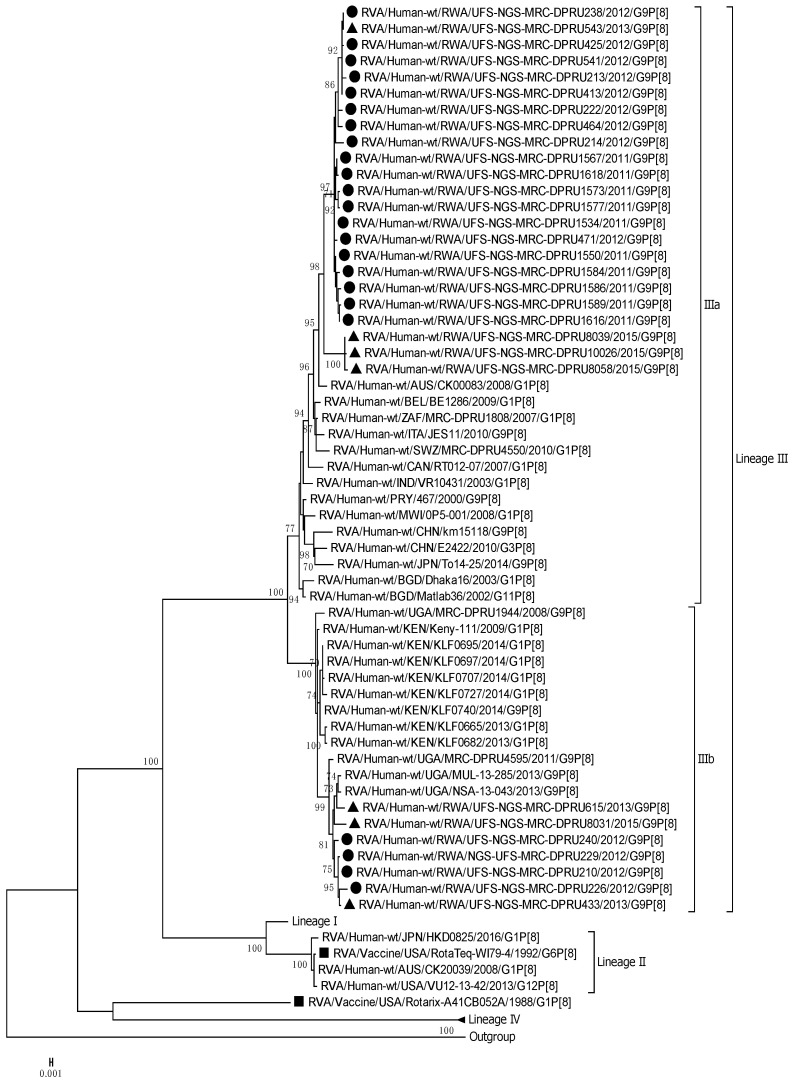
Maximum likelihood phylogenetic trees constructed from the nucleotide sequences of the Rwandan P[8] strains for the VP4 genome. Using 1000 bootstrap replicates, the branch support was thoroughly evaluated. All G9P[8] study strains clustered into Lineage III. The pre-vaccination study strains are indicated by a black circular shape, the post-vaccination study strains are indicated by a triangular shape, and the vaccine strains are indicated by a square shape.

The VP4 genome segment of the G9 Rwandan study strains was phylogenetically compared to four established lineages (I–IV) [31,32] (Figure 2). The P[8] sequences of the Rwandan G9P[8] study strains, together with global reference P[8] sequences, were segregated into four lineages. The P[8] study strains all clustered into lineage III, and this lineage comprised sequences primarily from East Africa, more specifically, Kenya and Uganda. Rwandan strains within lineage III were discovered to be highly linked, with nucleotide similarity ranging from 96% to 100%. The VP4 genome segment showed 96,6% to 100% nucleotide similarity within the different sub-clustering pattern of the three 2015 post-vaccination research strains. Several robust sub-lineages were defined within lineage III using a 70% threshold node. Thus, we identified that the Rwandan G9P[8] sequences clustered into sub-lineage IIIa and IIIb, respectively, within lineage III. Sub-lineage IIIa comprised 23 Rwandan G9 sequences, including three G9 sequences from 2015, and sub-lineage IIIb comprised seven Rwandan G9P[8] study strains. 

### 3.4. Sequence Analysis and Phylogenetic Analysis of the VP1-3, VP6, and NSP1-5 Genomes

The remaining nine genome segments, (VP1-3, VP6, and NSP1-5) (Supplementary Materials) together with the Rwandan G9 pre- and post-vaccination study strains, phylogenetically clustered closely with other selected reference global RVA strains. For these genome segments, the Rwandan G9P[8] study stains also clustered closely with reference strains originating from Eastern African regions, with nucleotide similarity ranges of 95.4% to 100%. 

The VP1 sequences were found to be closely related amongst each other, with a moderate nucleotide similarity range of 93.7% to 100%, and amongst the three sub-clustering post-vaccine strains, a 93.7% to 100% similarity range was reported. Within the VP2 gene sequences, the Rwandan study strains shared high nucleotide identities in a range of 95.8% to 100%. The three distinct post-vaccine strains, which sub-clustered closely together, demonstrated a nucleotide similarity range of 97.9% to 100% for the VP2 genome segment. A nucleotide similarity range of 94.2% to 100% was observed amongst the VP3 genome segment of the G9 study strains. A range of 96.9% to 100% was observed amongst the three post-vaccine strains, which were noticeably sub-clustered together. For the VP6 genome segment, the 30 Rwandan study strains, when compared to the RVA reference strains, were found to be closely related amongst each other, with a moderate nucleotide similarity range of 94.7% to 100%, and amongst the sub-clustering post-vaccine strains, a 94.7% to 100% similarity range was reported. 

The five non-structural proteins presented a diverse range of nucleotide similarities, firstly, with a nucleotide similarity range of 95.7% to 100% amongst the Rwandan G9 study strains for the NSP1 genome segment. The three distinct post-vaccine strains, which were sub-clustered closely together, demonstrated a nucleotide similarity range of 97.7% to 99.9% for this genome segment. The NSP2 genome of the 30 G9P[8] study strains presented a moderate nucleotide similarity range of 90.4% to 100%. A range of 97.5% to 100% was observed amongst the post-vaccine strains, which were noticeably sub-clustered together. For the NSP3 gene sequences, the pre- and post-vaccination study strains clustered closely together and revealed a shared nucleotide identity in the range of 92% to 100%, and amongst the three sub-clustering post-vaccine strains, a 98.9% to 100% similarity range was reported. Phylogenetically, the global RVA reference strains were compared to the NSP4 genome of 30 Rwandan G9P[8] study strains and found to have a distinct nucleotide similarity range of 81.5% to 100% amongst each other. The post-vaccine strains, which were sub-clustered distinctly together demonstrated a nucleotide similarity range of 97.1% to 100%. The NSP5 gene sequences of the 30 Rwandan study strains clustered closely together and demonstrated the highest level of similarity, with a range of 97.4% to 100%. A high range of 99.7% to 100% was observed amongst the three post-vaccine strains, which were strikingly sub-clustering together.

## 4. Discussion

This study analyzed 30 Rwandan G9P[8] study strains at the whole-genome level, revealing that all the G9 sequences from both the pre- and post-vaccination periods exhibited the pure Wa-like genotype constellation. Pure genome constellations originating from the same genotype are suggested to be a result of the co-evolution of optimal functioning proteins and epidemiological fitness [33,34]. This may also suggest that the pure nature of the constellation of these strains has contributed to them persisting in the population during the pre- and post-vaccination periods [34,35].

Phylogenetically, all 11 genome segments of the Rwandan G9P[8] study strains were highly similar and clustered closely together with the global reference RVA G9P[8] strains, and were further segregated into lineages that either consisted solely of pre- or post-vaccination strains, or that consisted of a mixture of the two vaccination periods. The emergence of defined lineages or sub-lineages is credited to diverse evolutionary mechanisms such as recombination, mutations, and reassortments [24,36,37].

A unique sub-clustering pattern was observed in three of the 2015 Rwandan G9P[8] post-vaccination study strains, which created a significant difference between the pre- and post-vaccination strains that are circulating in Rwanda. This may be due to the vaccine candidate being used, which failed to provide protection against the ever-changing virus and may not be as affective against these post-vaccination strains [38,39,40]. All 30 of the Rwandan G9 study strain sequences clustered into lineage III for the VP7 and VP4 genome segments, and furthermore, clustered into further defined sub-lineages [31]. The same has been reported in other studies in Africa and Asia, where both pre- and post-G9P[8] vaccination strains have clustered into lineage III, highlighting its predominance [41,42,43,44,45]. The G9 genotype typically clusters into lineage III, as all the Rwandan G9 study strains clustered into lineage III; this may demonstrate pre-existing G9 immunity in the population due to G9 strains being present previously.

Three post-vaccination Rwandan strains from 2015 distinctly sub-clustered together for all 11 genome segments. A close phylogenetic relationship was observed between the G9 study strains and reference sequences from the Eastern African regions, more specifically, Kenya and Uganda, suggesting co-circulation amongst these neighboring countries [46,47]. Due to the high nucleotide similarities between the Rwandan G9P[8] study strains and those strains from neighboring countries, this suggests local evolution. This underscores the importance of sharing epidemiological data amongst neighboring countries to monitor the circulation and emergence of strains, as rotavirus can readily disseminate throughout the population [46]. 

Sequence analysis of each gene segment revealed high nucleotide similarities (92.4–100%) for all gene segments except for the NSP2 and NSP4 genome segments, which presented a moderate nucleotide similarity range of 90.4% to 100%, and a diverse nucleotide similarity of 81.5% to 100%, respectively, when compared with the globally selected RVA G9P[8] reference strains. This may suggest that the G9 study sequences are related through evolutionary changes from a common ancestral sequence due to the high degree of nucleotide similarity amongst themselves [48].

This study identified amino acid differences in the neutralization epitope regions between the VP7 and VP4 study strains and the Rotarix^®^, RotaTeq^®^, Rotavac^®^, and Rotasiil^®^ vaccine strains. The Rotasiil^®^ and RotaVac^®^ vaccine strains were not included in the analysis of the VP4 neutralization epitope, as VP4 in these vaccine strains is of non-human origin [18,49]. In addition, the RotaTeq^®^ VP4 P[8] clustered in lineage II, and the VP4 P[8] of the Rwandan study strains clustered into lineage III, which translates to VP4 amino acid differences prior to vaccination. At position 113, 4/23 pre-vaccine strains contained aspartic acid. The 19 remaining pre-vaccine strains and the RotaTeq^®^ vaccine strain contained asparagine at that position. Post-vaccination, 3/7 study strains contained the N113D substitution, which could suggest vaccine pressure at this epitope, but additional data would be required to support this conclusion. The RotaTeq^®^ vaccine introduced in Rwanda is genotypically heterologous to VP7 G9, and this may be a factor in the dominance of the G9P[8] genotype before vaccine introduction, as it was circulating in 2012 and 2013 [50,51]. 

The observed amino acid differences included amino acid substitutions, as well as radical changes, meaning a change was observed in polarity or charge. These changes may play a role in the neuralization escape of the vaccine candidate for these strains and may contribute to the escape of host immunity, as these findings were specifically found post-vaccination introduction [52]. These changes may also contribute to the decreased antibody binding at those regions of the epitopes, which results in the epitope becoming inaccessible [53].

Due to the asymmetric nature and limited sample size of the presented dataset, a limitation to our study was observed, as a greater number of samples were sequenced pre- vaccination compared to samples that were sequenced from the post-vaccine era. Despite this limitation, we believe that this study provides significant insight into the evolution of G9P[8] strains circulating in Rwanda, which can be valuable in potentially predicating the vaccine’s impact in this region. 

In conclusion, our findings indicate that the Rwandan G9P[8] strains revealed a distinct sub-clustering pattern among post-vaccination 2015 study strains circulating in Rwanda, with changes at neutralization epitopes, which may play a role in neutralization escape from vaccine candidates for these strains. This emphasizes the need for continuous whole-genome surveillance to better understand the evolution and epidemiology of the G9P[8] strains post-vaccination, and to further assess the vaccine’s impact on circulating rotavirus strains in Rwanda.

## Figures and Tables

**Table 1:** Amino acid differences in the neutralizing epitope regions of the VP7 gene segment between Rwandan G9P[8] study strains and rotavirus vaccine strains. The colored shading is used to indicate amino acid differences that were observed. The array of colored shading of the amino acid residues for the vaccine strains represents changes that were observed when comparing the G9P[8] study strains to these vaccine strains.

		Neutralisation Epitopes																												
									7-1a									7-1b								7-2				
		87	91	94	96	97	98	99	100	104	123	125	129	130	291	201	211	212	213	238	242	143	145	146	147	148	190	217	221	264
**Vaccine strains**	RVA/Vaccine/USA/RotaTeq-WI79-9/1992/G1P75	**T**	**T**	**N**	**G**	**D**	**W**	**K**	**D**	**Q**	**S**	**V**	**V**	**D**	**K**	**Q**	**N**	**V**	**D**	**N**	**T**	**K**	**D**	**Q**	**S**	**L**	**S**	**M**	**N**	**G**
	RVA/Vaccine/USA/RotaTeq-WI79-9/1992/G2	**A**	**N**	**S**	**D**	**E**	**W**	**E**	**N**	**Q**	**D**	**T**	**M**	**N**	**K**	**Q**	**D**	**V**	**S**	**N**	**S**	**R**	**D**	**N**	**T**	**S**	**D**	**I**	**S**	**G**
	RVA/Vaccine/USA/RotaTeq-WI79-9/1992/G3	**T**	**T**	**N**	**N**	**S**	**W**	**K**	**D**	**Q**	**D**	**A**	**V**	**D**	**K**	**Q**	**D**	**A**	**N**	**K**	**D**	**K**	**D**	**A**	**T**	**L**	**S**	**E**	**A**	**G**
	RVA/Vaccine/USA/RotaTeq-WI79-9/1992/G4	**S**	**T**	**S**	**T**	**E**	**W**	**K**	**D**	**Q**	**N**	**L**	**I**	**D**	**K**	**Q**	**D**	**T**	**A**	**D**	**T**	**R**	**A**	**S**	**G**	**E**	**S**	**T**	**S**	**G**
	RVA/Vaccine/USA/Rotarix-A41CB052A/1988/G1P1A8	**T**	**T**	**N**	**G**	**E**	**W**	**K**	**D**	**Q**	**S**	**V**	**V**	**D**	**K**	**Q**	**N**	**V**	**D**	**N**	**T**	**K**	**D**	**Q**	**N**	**L**	**S**	**M**	**N**	**G**
	RVA/Vaccine/IND/Rotavac-116E/AG/G9P[11]	**I**	**T**	**G**	**T**	**E**	**W**	**K**	**G**	**Q**	**D**	**A**	**I**	**D**	**K**	**Q**	**N**	**T**	**A**	**D**	**N**	**K**	**N**	**S**	**T**	**L**	**S**	**E**	**N**	**G**
	RVA/Vaccine/IND/Rotasill-Au32/2016/G9	**A**	**T**	**G**	**T**	**E**	**W**	**K**	**D**	**Q**	**D**	**A**	**I**	**D**	**K**	**Q**	**N**	**T**	**A**	**D**	**T**	**K**	**D**	**S**	**T**	**L**	**S**	**E**	**S**	**G**
**Pre- vaccination strains**	**RVA/Human-wt/RWA/UFS-NGS-MRC-DPRU1618/2011/G9P[8]**	**T**	**T**	**G**	**T**	**E**	**W**	**K**	**G**	**Q**	**D**	**A**	**I**	**D**	**K**	**Q**	**N**	**T**	**A**	**D**	**N**	**K**	**D**	**S**	**T**	**L**	**S**	**E**	**S**	**G**
	**RVA/Human-wt/RWA/UFS-NGS-MRC-DPRU1616/2011/G9P[8]**	**T**	**T**	**G**	**T**	**E**	**W**	**K**	**G**	**Q**	**D**	**A**	**I**	**D**	**K**	**Q**	**N**	**T**	**A**	**D**	**N**	**K**	**D**	**S**	**T**	**L**	**S**	**E**	**S**	**G**
	**RVA/Human-wt/RWA/UFS-NGS-MRC-DPRU1589/2011/G9P[8]**	**T**	**T**	**G**	**T**	**E**	**W**	**K**	**G**	**Q**	**D**	**A**	**I**	**D**	**K**	**Q**	**N**	**T**	**A**	**D**	**N**	**K**	**D**	**S**	**T**	**L**	**S**	**E**	**S**	**G**
	**RVA/Human-wt/RWA/UFS-NGS-MRC-DPRU1586/2011/G9P[8]**	**T**	**T**	**G**	**T**	**E**	**W**	**K**	**G**	**Q**	**D**	**A**	**I**	**D**	**K**	**Q**	**N**	**T**	**A**	**D**	**N**	**K**	**D**	**S**	**T**	**L**	**S**	**E**	**S**	**G**
	**RVA/Human-wt/RWA/UFS-NGS-MRC-DPRU1584/2011/G9P[8]**	**T**	**T**	**G**	**T**	**E**	**W**	**K**	**G**	**Q**	**D**	**A**	**I**	**D**	**K**	**Q**	**N**	**T**	**A**	**D**	**N**	**K**	**D**	**S**	**T**	**L**	**S**	**E**	**S**	**G**
	**RVA/Human-wt/RWA/UFS-NGS-MRC-DPRU1577/2011/G9P[8]**	**T**	**T**	**G**	**T**	**E**	**W**	**K**	**G**	**Q**	**D**	**A**	**I**	**D**	**K**	**Q**	**N**	**T**	**A**	**D**	**N**	**K**	**D**	**S**	**T**	**L**	**S**	**E**	**S**	**G**
	**RVA/Human-wt/RWA/UFS-NGS-MRC-DPRU1573/2011/G9P[8]**	**T**	**T**	**G**	**T**	**E**	**W**	**K**	**G**	**Q**	**D**	**A**	**I**	**D**	**K**	**Q**	**N**	**T**	**A**	**D**	**N**	**K**	**D**	**S**	**T**	**L**	**S**	**E**	**S**	**G**
	**RVA/Human-wt/RWA/UFS-NGS-MRC-DPRU1567/2011/G9P[8]**	**T**	**T**	**G**	**T**	**E**	**W**	**K**	**G**	**Q**	**D**	**A**	**I**	**D**	**K**	**Q**	**N**	**T**	**A**	**D**	**N**	**K**	**D**	**S**	**T**	**L**	**S**	**E**	**S**	**G**
	**RVA/Human-wt/RWA/UFS-NGS-MRC-DPRU1550/2011/G9P[8]**	**T**	**T**	**G**	**T**	**E**	**W**	**K**	**G**	**Q**	**D**	**A**	**I**	**D**	**K**	**Q**	**N**	**T**	**A**	**D**	**N**	**K**	**D**	**S**	**T**	**L**	**S**	**E**	**S**	**G**
	**RVA/Human-wt/RWA/UFS-NGS-MRC-DPRU1534/2011/G9P[8]**	**T**	**T**	**G**	**T**	**E**	**W**	**K**	**G**	**Q**	**D**	**A**	**I**	**D**	**K**	**Q**	**N**	**T**	**A**	**D**	**N**	**K**	**D**	**S**	**T**	**L**	**S**	**E**	**S**	**G**
	**RVA/Human-wt/RWA/UFS-NGS-MRC-DPRU541/2012/G9P[8]**	**T**	**T**	**G**	**T**	**E**	**W**	**K**	**G**	**Q**	**D**	**A**	**I**	**D**	**K**	**Q**	**N**	**T**	**A**	**D**	**N**	**K**	**D**	**S**	**T**	**L**	**S**	**E**	**S**	**G**
	**RVA/Human-wt/RWA/UFS-NGS-MRC-DPRU471/2012/G9P[8]**	**T**	**T**	**G**	**T**	**E**	**W**	**K**	**G**	**Q**	**D**	**A**	**I**	**D**	**K**	**Q**	**N**	**T**	**A**	**D**	**N**	**K**	**D**	**S**	**T**	**L**	**S**	**E**	**S**	**G**
	**RVA/Human-wt/RWA/UFS-NGS-MRC-DPRU464/2012/G9P[8]**	**T**	**T**	**G**	**T**	**E**	**W**	**K**	**G**	**Q**	**D**	**A**	**I**	**D**	**K**	**Q**	**N**	**T**	**A**	**D**	**N**	**K**	**D**	**S**	**T**	**L**	**S**	**E**	**S**	**G**
	**RVA/Human-wt/RWA/UFS-NGS-MRC-DPRU425/2012/G9P[8]**	**T**	**T**	**G**	**T**	**E**	**W**	**K**	**G**	**Q**	**D**	**A**	**I**	**D**	**K**	**Q**	**N**	**T**	**A**	**D**	**N**	**K**	**D**	**S**	**T**	**L**	**S**	**E**	**S**	**G**
	**RVA/Human-wt/RWA/UFS-NGS-MRC-DPRU413/2012/G9P[8]**	**T**	**T**	**G**	**T**	**E**	**W**	**K**	**G**	**Q**	**D**	**A**	**I**	**D**	**K**	**Q**	**N**	**T**	**A**	**D**	**N**	**K**	**D**	**S**	**T**	**L**	**S**	**E**	**S**	**G**
	**RVA/Human-wt/RWA/UFS-NGS-MRC-DPRU240/2012/G9P[8]**	**T**	**T**	**G**	**T**	**E**	**W**	**K**	**D**	**Q**	**D**	**A**	**I**	**D**	**K**	**Q**	**N**	**T**	**A**	**D**	**N**	**K**	**D**	**S**	**T**	**L**	**S**	**E**	**S**	**G**
	**RVA/Human-wt/RWA/UFS-NGS-MRC-DPRU238/2012/G9P[8]**	**T**	**T**	**G**	**T**	**E**	**W**	**K**	**G**	**Q**	**D**	**A**	**I**	**D**	**K**	**Q**	**N**	**T**	**A**	**D**	**N**	**K**	**D**	**S**	**T**	**L**	**S**	**E**	**S**	**G**
	**RVA/Human-wt/RWA/UFS-NGS-MRC-DPRU226/2012/G9P[8]**	**T**	**T**	**G**	**T**	**E**	**W**	**K**	**D**	**Q**	**D**	**A**	**I**	**D**	**K**	**Q**	**N**	**T**	**A**	**D**	**N**	**K**	**D**	**S**	**T**	**L**	**S**	**E**	**S**	**G**
	**RVA/Human-wt/RWA/UFS-NGS-MRC-DPRU222/2012/G9P[8]**	**T**	**T**	**G**	**T**	**E**	**W**	**K**	**G**	**Q**	**D**	**A**	**I**	**D**	**K**	**Q**	**N**	**T**	**A**	**D**	**N**	**K**	**D**	**S**	**T**	**L**	**S**	**E**	**S**	**G**
	**RVA/Human-wt/RWA/UFS-NGS-MRC-DPRU214/2012/G9P[8]**	**T**	**T**	**G**	**T**	**E**	**W**	**K**	**G**	**Q**	**D**	**A**	**I**	**D**	**K**	**Q**	**N**	**T**	**A**	**D**	**N**	**K**	**D**	**S**	**T**	**L**	**S**	**E**	**S**	**G**
	**RVA/Human-wt/RWA/UFS-NGS-MRC-DPRU213/2012/G9P[8]**	**T**	**T**	**G**	**T**	**E**	**W**	**K**	**G**	**Q**	**D**	**A**	**I**	**D**	**K**	**Q**	**N**	**T**	**A**	**D**	**N**	**K**	**D**	**S**	**T**	**L**	**S**	**E**	**S**	**G**
	**RVA/Human-wt/RWA/UFS-NGS-MRC-DPRU210/2012/G9P[8]**	**T**	**T**	**G**	**T**	**E**	**W**	**K**	**D**	**Q**	**D**	**A**	**I**	**D**	**K**	**Q**	**N**	**T**	**A**	**D**	**N**	**K**	**D**	**S**	**T**	**L**	**S**	**E**	**S**	**G**
	**RVA/Human-wt/RWA/NGS-UFS-MRC-DPRU229/2012/G9P[8]**	**T**	**T**	**G**	**T**	**E**	**W**	**K**	**D**	**Q**	**D**	**A**	**I**	**D**	**K**	**Q**	**N**	**T**	**A**	**D**	**N**	**K**	**D**	**S**	**T**	**L**	**S**	**E**	**S**	**G**
**Post- vaccination strains**	**RVA/Human-wt/RWA/UFS-NGS-MRC-DPRU615/2013/G9P[8]**	**T**	**T**	**G**	**T**	**E**	**W**	**K**	**D**	**Q**	**D**	**A**	**I**	**D**	**K**	**Q**	**N**	**T**	**A**	**D**	**N**	**K**	**D**	**S**	**T**	**L**	**S**	**E**	**S**	**G**
	**RVA/Human-wt/RWA/UFS-NGS-MRC-DPRU543/2013/G9P[8]**	**T**	**T**	**G**	**T**	**E**	**W**	**K**	**G**	**Q**	**D**	**A**	**I**	**D**	**K**	**Q**	**N**	**T**	**A**	**D**	**N**	**K**	**D**	**S**	**T**	**L**	**S**	**E**	**S**	**G**
	**RVA/Human-wt/RWA/UFS-NGS-MRC-DPRU433/2013/G9P[8]**	**T**	**T**	**G**	**T**	**E**	**W**	**K**	**D**	**Q**	**D**	**A**	**I**	**D**	**K**	**Q**	**N**	**T**	**A**	**D**	**N**	**K**	**D**	**S**	**T**	**L**	**S**	**E**	**S**	**G**
	**RVA/Human-wt/RWA/UFS-NGS-MRC-DPRU8058/2015/G9P[8]**	**T**	**T**	**G**	**T**	**E**	**W**	**K**	**D**	**Q**	**D**	**A**	**I**	**D**	**K**	**Q**	**N**	**T**	**A**	**D**	**S**	**K**	**D**	**S**	**T**	**L**	**S**	**E**	**S**	**G**
	**RVA/Human-wt/RWA/UFS-NGS-MRC-DPRU8039/2015/G9P[8]**	**T**	**T**	**G**	**T**	**E**	**W**	**K**	**D**	**Q**	**D**	**A**	**I**	**D**	**K**	**Q**	**N**	**T**	**A**	**D**	**S**	**K**	**D**	**S**	**T**	**L**	**S**	**E**	**S**	**G**
	**RVA/Human-wt/RWA/UFS-NGS-MRC-DPRU10026/2015/G9P[8]**	**T**	**T**	**G**	**T**	**E**	**W**	**K**	**D**	**Q**	**D**	**A**	**I**	**D**	**K**	**Q**	**N**	**T**	**A**	**D**	**S**	**K**	**D**	**S**	**T**	**L**	**S**	**E**	**S**	**G**
	**RVA/Human-wt/RWA/UFS-NGS-MRC-DPRU8031/2015/G9P[8]**	**T**	**T**	**G**	**A**	**E**	**W**	**K**	**D**	**Q**	**D**	**A**	**I**	**D**	**K**	**Q**	**N**	**T**	**A**	**D**	**N**	**K**	**D**	**S**	**T**	**L**	**S**	**E**	**S**	**G**

**Table 2:** Amino acid differences in the neutralizing epitope regions of the VP4 gene segment between Rwandan G9P[8] study strains and two of the rotavirus vaccine strains. The colored shading is used to indicate amino acid differences that were observed. The array of colored shading of the amino acid residues for the vaccine strains represents changes that were observed when comparing the G9P[8] study strains to these vaccine strains.

		Neutralisation Epitopes																																			
		8-1											8-2		8-3								8-4			5-1								5-2	5-3	5-4	5-5
		100	146	148	150	188	190	192	193	194	###	196	180	183	113	114	###	125	131	132	133	135	87	88	89	###	###	388	393	394	398	440	441	434	459	429	306
**Vaccine strains**	RVA/Vaccine/USA/Rotarix-A41CB052A/1988/G1P1A8	**D**	**S**	**Q**	**E**	**S**	**T**	**N**	**L**	**N**	**N**	**I**	**T**	**A**	**N**	**P**	**V**	**S**	**S**	**N**	**D**	**N**	**N**	**T**	**N**	**Y**	**F**	**I**	**W**	**P**	**G**	**R**	**T**	**P**	**E**	**L**	**R**
	RVA/Vaccine/USA/RotaTeq-WI79-4/1992/G6P1A8	**D**	**S**	**Q**	**E**	**S**	**T**	**N**	**L**	**N**	**D**	**I**	**T**	**A**	**N**	**P**	**V**	**N**	**R**	**N**	**D**	**D**	**N**	**T**	**N**	**Y**	**F**	**L**	**W**	**P**	**G**	**R**	**T**	**P**	**E**	**L**	**R**
**Pre- vaccination strains**	**RVA/Human-wt/RWA/UFS-NGS-MRC-DPRU1534/2011/G9P8**	**D**	**S**	**Q**	**D**	**S**	**T**	**N**	**L**	**N**	**G**	**I**	**T**	**A**	**N**	**P**	**V**	**N**	**R**	**N**	**D**	**D**	**N**	**T**	**N**	**Y**	**F**	**I**	**W**	**P**	**G**	**R**	**T**	**P**	**E**	**L**	**R**
	**RVA/Human-wt/RWA/UFS-NGS-MRC-DPRU1550/2011/G9P8**	**D**	**S**	**Q**	**D**	**S**	**T**	**N**	**L**	**N**	**G**	**I**	**T**	**A**	**N**	**P**	**V**	**N**	**R**	**N**	**D**	**D**	**N**	**T**	**N**	**Y**	**F**	**I**	**W**	**P**	**G**	**R**	**T**	**P**	**E**	**L**	**R**
	**RVA/Human-wt/RWA/UFS-NGS-MRC-DPRU1567/2011/G9P8**	**D**	**S**	**Q**	**D**	**S**	**T**	**N**	**L**	**N**	**G**	**I**	**T**	**A**	**N**	**P**	**V**	**N**	**R**	**N**	**D**	**D**	**N**	**T**	**N**	**Y**	**F**	**I**	**W**	**P**	**G**	**R**	**T**	**P**	**E**	**L**	**R**
	**RVA/Human-wt/RWA/UFS-NGS-MRC-DPRU1573/2011/G9P8**	**D**	**S**	**Q**	**D**	**S**	**T**	**N**	**L**	**N**	**G**	**I**	**T**	**A**	**N**	**P**	**V**	**N**	**R**	**N**	**D**	**D**	**N**	**T**	**N**	**Y**	**F**	**I**	**W**	**P**	**G**	**R**	**T**	**P**	**E**	**L**	**R**
	**RVA/Human-wt/RWA/UFS-NGS-MRC-DPRU1577/2011/G9P8**	**D**	**S**	**Q**	**D**	**S**	**T**	**N**	**L**	**N**	**G**	**I**	**T**	**A**	**N**	**P**	**V**	**N**	**R**	**N**	**D**	**D**	**N**	**T**	**N**	**Y**	**F**	**I**	**W**	**P**	**G**	**R**	**T**	**P**	**E**	**L**	**R**
	**RVA/Human-wt/RWA/UFS-NGS-MRC-DPRU1584/2011/G9P8**	**D**	**S**	**Q**	**D**	**S**	**T**	**N**	**L**	**N**	**G**	**I**	**T**	**A**	**N**	**P**	**V**	**N**	**R**	**N**	**D**	**D**	**N**	**T**	**N**	**Y**	**F**	**I**	**W**	**P**	**G**	**R**	**T**	**P**	**E**	**L**	**R**
	**RVA/Human-wt/RWA/UFS-NGS-MRC-DPRU1586/2011/G9P8**	**D**	**S**	**Q**	**D**	**S**	**T**	**N**	**L**	**N**	**G**	**L**	**T**	**A**	**N**	**P**	**V**	**N**	**R**	**N**	**D**	**D**	**N**	**T**	**N**	**Y**	**F**	**I**	**W**	**P**	**G**	**R**	**T**	**P**	**E**	**L**	**R**
	**RVA/Human-wt/RWA/UFS-NGS-MRC-DPRU1589/2011/G9P8**	**D**	**S**	**Q**	**D**	**S**	**T**	**N**	**L**	**N**	**G**	**I**	**T**	**A**	**N**	**P**	**V**	**N**	**R**	**N**	**D**	**D**	**N**	**T**	**N**	**Y**	**F**	**I**	**W**	**P**	**G**	**R**	**T**	**P**	**E**	**L**	**R**
	**RVA/Human-wt/RWA/UFS-NGS-MRC-DPRU1616/2011/G9P8**	**D**	**S**	**Q**	**D**	**S**	**T**	**N**	**L**	**N**	**G**	**I**	**T**	**A**	**N**	**P**	**V**	**N**	**R**	**N**	**D**	**D**	**N**	**T**	**N**	**Y**	**F**	**I**	**W**	**P**	**G**	**R**	**T**	**P**	**E**	**L**	**R**
	**RVA/Human-wt/RWA/UFS-NGS-MRC-DPRU1618/2011/G9P8**	**D**	**S**	**Q**	**D**	**S**	**T**	**N**	**L**	**N**	**G**	**I**	**T**	**A**	**N**	**P**	**V**	**N**	**R**	**N**	**D**	**D**	**N**	**T**	**N**	**Y**	**F**	**I**	**W**	**P**	**G**	**R**	**T**	**P**	**E**	**L**	**R**
	**RVA/Human-wt/RWA/UFS-NGS-MRC-DPRU210/2012/G9P8**	**D**	**S**	**Q**	**D**	**S**	**T**	**N**	**L**	**N**	**G**	**I**	**T**	**A**	**D**	**P**	**V**	**N**	**R**	**N**	**D**	**D**	**N**	**T**	**N**	**Y**	**F**	**I**	**W**	**P**	**G**	**R**	**T**	**P**	**E**	**L**	**R**
	**RVA/Human-wt/RWA/UFS-NGS-MRC-DPRU213/2012/G9P8**	**D**	**S**	**Q**	**D**	**S**	**T**	**N**	**L**	**N**	**G**	**I**	**T**	**A**	**N**	**P**	**V**	**N**	**R**	**N**	**D**	**D**	**N**	**T**	**N**	**Y**	**F**	**I**	**W**	**P**	**G**	**R**	**T**	**P**	**E**	**L**	**R**
	**RVA/Human-wt/RWA/UFS-NGS-MRC-DPRU214/2012/G9P8**	**D**	**S**	**Q**	**D**	**S**	**T**	**N**	**L**	**N**	**G**	**I**	**T**	**A**	**N**	**P**	**V**	**N**	**R**	**N**	**D**	**D**	**N**	**T**	**N**	**Y**	**F**	**I**	**W**	**P**	**G**	**R**	**T**	**P**	**E**	**L**	**R**
	**RVA/Human-wt/RWA/UFS-NGS-MRC-DPRU222/2012/G9P8**	**D**	**S**	**Q**	**D**	**S**	**T**	**N**	**L**	**N**	**G**	**I**	**T**	**A**	**N**	**P**	**V**	**N**	**R**	**N**	**D**	**D**	**N**	**T**	**N**	**Y**	**F**	**I**	**W**	**P**	**G**	**R**	**T**	**P**	**E**	**L**	**R**
	**RVA/Human-wt/RWA/NGS-UFS-MRC-DPRU229/2012/G9P8**	**D**	**S**	**Q**	**D**	**S**	**T**	**N**	**L**	**N**	**G**	**I**	**T**	**A**	**D**	**P**	**V**	**N**	**R**	**N**	**D**	**D**	**N**	**T**	**N**	**Y**	**F**	**I**	**W**	**P**	**G**	**R**	**T**	**P**	**E**	**L**	**R**
	**RVA/Human-wt/RWA/UFS-NGS-MRC-DPRU226/2012/G9P8**	**D**	**S**	**Q**	**D**	**S**	**T**	**N**	**L**	**N**	**G**	**I**	**T**	**A**	**D**	**P**	**V**	**N**	**R**	**N**	**D**	**D**	**N**	**T**	**N**	**Y**	**F**	**I**	**W**	**P**	**G**	**R**	**T**	**P**	**E**	**L**	**R**
	**RVA/Human-wt/RWA/UFS-NGS-MRC-DPRU238/2012/G9P8**	**D**	**S**	**Q**	**D**	**S**	**T**	**N**	**L**	**N**	**G**	**I**	**T**	**A**	**N**	**P**	**V**	**N**	**R**	**N**	**D**	**D**	**N**	**T**	**N**	**Y**	**F**	**I**	**W**	**P**	**G**	**R**	**T**	**P**	**E**	**L**	**R**
	**RVA/Human-wt/RWA/UFS-NGS-MRC-DPRU240/2012/G9P8**	**D**	**S**	**Q**	**D**	**S**	**T**	**N**	**L**	**N**	**G**	**I**	**T**	**A**	**D**	**P**	**V**	**N**	**R**	**N**	**D**	**D**	**N**	**T**	**N**	**Y**	**F**	**I**	**W**	**P**	**G**	**R**	**T**	**P**	**E**	**L**	**R**
	**RVA/Human-wt/RWA/UFS-NGS-MRC-DPRU413/2012/G9P8**	**D**	**S**	**Q**	**D**	**S**	**T**	**N**	**L**	**N**	**G**	**I**	**T**	**A**	**N**	**P**	**V**	**N**	**R**	**N**	**D**	**D**	**N**	**T**	**N**	**Y**	**F**	**I**	**W**	**P**	**G**	**R**	**T**	**P**	**E**	**L**	**R**
	**RVA/Human-wt/RWA/UFS-NGS-MRC-DPRU425/2012/G9P8**	**D**	**S**	**Q**	**D**	**S**	**T**	**N**	**L**	**N**	**G**	**I**	**T**	**A**	**N**	**P**	**V**	**N**	**R**	**N**	**D**	**D**	**N**	**T**	**N**	**Y**	**F**	**I**	**W**	**P**	**G**	**R**	**T**	**P**	**E**	**L**	**R**
	**RVA/Human-wt/RWA/UFS-NGS-MRC-DPRU464/2012/G9P8**	**D**	**S**	**Q**	**D**	**S**	**T**	**N**	**L**	**N**	**G**	**I**	**T**	**A**	**N**	**P**	**V**	**N**	**R**	**N**	**D**	**D**	**N**	**T**	**N**	**Y**	**F**	**I**	**W**	**P**	**G**	**R**	**T**	**P**	**E**	**L**	**R**
	**RVA/Human-wt/RWA/UFS-NGS-MRC-DPRU471/2012/G9P8**	**D**	**S**	**Q**	**D**	**S**	**T**	**N**	**L**	**N**	**G**	**I**	**T**	**A**	**N**	**P**	**V**	**N**	**R**	**N**	**D**	**D**	**N**	**T**	**N**	**Y**	**F**	**I**	**W**	**P**	**G**	**R**	**T**	**P**	**E**	**L**	**R**
	**RVA/Human-wt/RWA/UFS-NGS-MRC-DPRU541/2012/G9P8**	**D**	**S**	**Q**	**D**	**S**	**T**	**N**	**L**	**N**	**G**	**I**	**T**	**A**	**N**	**P**	**V**	**N**	**R**	**N**	**D**	**D**	**N**	**T**	**N**	**Y**	**F**	**I**	**W**	**P**	**G**	**R**	**T**	**P**	**E**	**L**	**R**
**Post- vaccination strains**	**RVA/Human-wt/RWA/UFS-NGS-MRC-DPRU543/2013/G9P8**	**D**	**S**	**Q**	**D**	**S**	**T**	**N**	**L**	**N**	**G**	**I**	**T**	**A**	**N**	**P**	**V**	**N**	**R**	**N**	**D**	**D**	**N**	**T**	**N**	**Y**	**F**	**I**	**W**	**P**	**G**	**R**	**T**	**P**	**E**	**L**	**R**
	**RVA/Human-wt/RWA/UFS-NGS-MRC-DPRU433/2013/G9P8**	**D**	**S**	**Q**	**D**	**S**	**T**	**N**	**L**	**N**	**G**	**I**	**T**	**A**	**D**	**P**	**V**	**N**	**R**	**N**	**D**	**D**	**N**	**T**	**N**	**Y**	**F**	**I**	**W**	**P**	**G**	**R**	**T**	**P**	**E**	**L**	**R**
	**RVA/Human-wt/RWA/UFS-NGS-MRC-DPRU615/2013/G9P8**	**D**	**S**	**Q**	**D**	**S**	**T**	**N**	**L**	**N**	**S**	**I**	**T**	**A**	**D**	**P**	**V**	**N**	**R**	**N**	**D**	**D**	**N**	**T**	**N**	**Y**	**F**	**I**	**W**	**P**	**G**	**R**	**T**	**P**	**E**	**L**	**R**
	**RVA/Human-wt/RWA/UFS-NGS-MRC-DPRU8031/2015/G9P8**	**D**	**S**	**Q**	**D**	**S**	**T**	**N**	**L**	**N**	**G**	**I**	**T**	**A**	**D**	**P**	**V**	**N**	**R**	**N**	**D**	**D**	**N**	**T**	**N**	**Y**	**F**	**I**	**W**	**P**	**G**	**R**	**T**	**P**	**E**	**L**	**R**
	**RVA/Human-wt/RWA/UFS-NGS-MRC-DPRU8039/2015/G9P8**	**D**	**S**	**Q**	**D**	**S**	**T**	**N**	**L**	**N**	**G**	**I**	**T**	**A**	**N**	**P**	**V**	**N**	**R**	**N**	**D**	**D**	**N**	**T**	**N**	**Y**	**F**	**I**	**W**	**P**	**G**	**R**	**T**	**P**	**E**	**L**	**R**
	**RVA/Human-wt/RWA/UFS-NGS-MRC-DPRU8058/2015/G9P8**	**D**	**S**	**Q**	**D**	**S**	**T**	**N**	**L**	**N**	**G**	**I**	**T**	**A**	**N**	**P**	**V**	**N**	**R**	**N**	**D**	**D**	**N**	**T**	**N**	**Y**	**F**	**I**	**W**	**P**	**G**	**R**	**T**	**P**	**E**	**L**	**R**
	**RVA/Human-wt/RWA/UFS-NGS-MRC-DPRU10026/2015/G9P8**	**D**	**S**	**Q**	**D**	**S**	**T**	**N**	**L**	**N**	**G**	**I**	**T**	**A**	**N**	**P**	**V**	**N**	**R**	**N**	**D**	**D**	**N**	**T**	**N**	**Y**	**F**	**I**	**W**	**P**	**G**	**R**	**T**	**P**	**E**	**L**	**R**

## Data Availability

All of the gene sequences in this G9P[8] Rwandan study were submitted to the NCBI GenBank database under accession numbers OR401005-OR401334, and are included in Supplementary Material S1.

## Supplementary Materials

The following supporting information can be downloaded at: https://www.mdpi.com/article/10.3390/v15122321/s1, Supplementary Material S1: Accession numbers of the sequences generated in this study of Rwandan G9P[8] strains and reference sequences used throughout the study; Supplementary Material S2: Maximum likelihood trees generated in this G9P[8] study; Supplementary Material S3: Nucleotide similarity analysis for all genome segments in the Rwandan G9P[8] study.

## References

[1] Walker C.L.F., Rudan I., Liu L., Nair H., Theodoratou E., Bhutta Z.A., O’Brien K.L., Campbell H., Black R.E. (2013). Global burden of childhood pneumonia and diarrhoea. Lancet.

[2] Parashar U.D., Gibson C.J., Bresee J.S., Glass R.I. (2006). Rotavirus and severe childhood diarrhea. Emerg. Infect. Dis..

[3] Troeger C., Khalil I.A., Rao P.C., Cao S., Blacker B.F., Ahmed T., Armah G., Bines J.E., Brewer T.G., Colombara D.V. (2018). Rotavirus vaccination and the global burden of rotavirus diarrhea among children younger than 5 years. JAMA Pediatr..

[4] CDC (2003). Managing Acute Gastroenteritis among Children: Oral Rehydration, Maintenance, and Nutritional Therapy [WWW Document]. https://www.cdc.gov/mmwr/preview/mmwrhtml/rr5216a1.htm.

[5] Madhi S.A., Cunliffe N.A., Steele D., Witte D., Kirsten M., Louw C. (2010). Effect of human rotavirus vaccine on severe diarrhea in African infants. New Engl. J. Med..

[6] Kirkwood C.D., Steele A.D. (2018). Rotavirus vaccines in China: Improvement still required. JAMA Netw. Open.

[7] Vesikari T. (2016). Success of rotavirus vaccination in Africa: Good news and remaining questions. Lancet Glob. Health.

[8] Gatera M., Bhatt S., Ngabo F., Utamuliza M., Sibomana H., Karema C., Mugeni C., Nutt C.T., Nsanzimana S., Wagner C.M. (2016). Successive introduction of four new vaccines in Rwanda: High coverage and rapid scale up of Rwanda’s expanded immunization program from 2009 to 2013. Vaccine.

[9] Sibomana H., Rugambwa C., Sayinzoga F., Iraguha G., Uwimana J. (2018). Impact of routine rotavirus vaccination on all-cause and rotavirus hospitalizations during the first four years following vaccine introduction in Rwanda. Vaccine.

[10] Matthijnssens J., Ciarlet M., Rahman M., Attoui H., Bányai K., Estes M.K., Gentsch J.R., Iturriza-Gómara M., Kirkwood C.D., Martella V. (2008). Recommendations for the classification of group A rotaviruses using all 11 genomic RNA segments. Arch. Virol..

[11] Patton J.T., Vasquez-Del Carpio R., Tortorici M.A., Taraporewala Z.F. (2006). Coupling of rotavirus genome replication and capsid assembly. Adv. Virus Res..

[12] Estes M.K., Cohen J.E.A.N. (1989). Rotavirus gene structure and function. Microbiol. Rev..

[13] Moure U.A.E., Banga-Mingo V., Gody J.C., Mwenda J.M., Fandema J., Waku-Kouomou D., Manengu C., Koyazegbe T.D.A., Esona M.D., Bowen M.D. (2018). Emergence of G12 and G9 rotavirus genotypes in the Central African Republic, January 2014 to February 2016. BMC Res. Notes.

[14] Zhou X., Wang Y.H., Pang B.B., Chen N., Kobayashi N. (2020). Surveillance of Human Rotavirus in Wuhan, China (2011–2019): Predominance of G9P [8] and Emergence of G12. Pathogens.

[15] Mwenda J.M., Ntoto K.M., Abebe A., Enweronu-Laryea C., Amina I., Mchomvu J., Kisakye A., Mpabalwani E.M., Pazvakavambwa I., Armah G.E. (2010). Burden and epidemiology of rotavirus diarrhea in selected African countries: Preliminary results from the African Rotavirus Surveillance Network. J. Infect. Dis..

[16] Nyaga M.M., Jere K.C., Peenze I., Mlera L., van Dijk A.A., Seheri M.L., Mphahlele M.J. (2013). Sequence analysis of the whole genomes of five African human G9 rotavirus strains. Infect. Genet. Evol..

[17] Doro R., László B., Martella V., Leshem E., Gentsch J., Parashar U., Bányai K. (2014). Review of global rotavirus strain prevalence data from six years post vaccine licensure surveillance: Is there evidence of strain selection from vaccine pressure?. Infect. Genet. Evol..

[18] Sadiq A., Bostan N., Khan J., Aziz A. (2022). Effect of rotavirus genetic diversity on vaccine impact. Rev. Med. Virol..

[19] Seheri L.M., Magagula N.B., Peenze I., Rakau K., Ndadza A., Mwenda J.M., Weldegebriel G., Steele A.D., Mphahlele M.J. (2018). Rotavirus strain diversity in Eastern and Southern African countries before and after vaccine introduction. Vaccine.

[20] Kabayiza J.C., Nilsson S., Andersson M. (2023). Rotavirus infections and their genotype distribution in Rwanda before and after the introduction of rotavirus vaccination. PLoS ONE.

[21] Burnett E., Parashar U.D., Tate J.E. (2020). Real-world effectiveness of rotavirus vaccines, 2006–2019: A literature review and meta-analysis. Lancet Glob. Health.

[22] Steele A.D., Victor J.C., Carey M.E., Tate J.E., Atherly D.E., Pecenka C., Diaz Z., Parashar U.D., Kirkwood C.D. (2019). Experiences with rotavirus vaccines: Can we improve rotavirus vaccine impact in developing countries?. Hum. Vaccines Immunother..

[23] Velasquez D.E., Parashar U., Jiang B. (2018). Decreased performance of live attenuated, oral rotavirus vaccines in low-income settings: Causes and contributing factors. Expert Rev. Vaccines.

[24] Rasebotsa S., Uwimana J., Mogotsi M.T., Rakau K., Magagula N.B., Seheri M.L., Mwenda J.M., Mphahlele M.J., Sabiu S., Mihigo R. (2021). Whole-genome analyses identifies multiple reassortant rotavirus strains in Rwanda post-vaccine introduction. Viruses.

[25] Mwangi P.N., Mogotsi M.T., Rasebotsa S.P., Seheri M.L., Mphahlele M.J., Ndze V.N., Dennis F.E., Jere K.C., Nyaga M.M. (2020). Uncovering the first atypical ds-1-like g1p [8] rotavirus strains that circulated during pre-rotavirus vaccine introduction era in South Africa. Pathogens.

[26] Nyaga M.M., Tan Y., Seheri M.L., Halpin R.A., Akopov A., Stucker K.M., Fedorova N.B., Shrivastava S., Steele A.D., Mwenda J.M. (2018). Whole-genome sequencing and analyses identify high genetic heterogeneity, diversity and endemicity of rotavirus genotype P [6] strains circulating in Africa. Infect. Genet. Evol..

[27] Andrews S., Krueger F., Segonds-Pichon A., Biggins L., Krueger C., Wingett S. (2010). A Quality Control Tool for High Throughput Sequence Data, 370.

[28] Kearse M., Moir R., Wilson A., Stones-Havas S., Cheung M., Sturrock S., Buxton S., Cooper A., Markowitz S., Duran C. (2012). Geneious Basic: An integrated and extendable desktop software platform for the organisation and analysis of sequence data. Bioinformatics.

[29] Aoki S.T., Settembre E.C., Trask S.D., Greenberg H.B., Harrison S.C., Dormitzer P.R. (2009). Structure of rotavirus outer-layer protein VP7 bound with a neutralizing Fab. Science.

[30] Dormitzer P.R., Sun Z.Y.J., Wagner G., Harrison S.C. (2002). The rhesus rotavirus VP4 sialic acid binding domain has a galectin fold with a novel carbohydrate binding site. EMBO J..

[31] Gupta S., Tiku V.R., Gauhar M., Khatoon K., Ray P. (2021). Genetic diversity of G9 rotavirus strains circulating among diarrheic children in North India: A comparison with 116E rotavirus vaccine strain. Vaccine.

[32] Yan N., Tang C., Kan R., Feng F., Yue H. (2019). Genome analysis of a G9P [23] group A rotavirus isolated from a dog with diarrhea in China. Infect. Genet. Evol..

[33] Nyaga M.M., Jere K.C., Esona M.D., Seheri M.L., Stucker K.M., Halpin R.A., Akopov A., Stockwell T.B., Peenze I., Diop A. (2015). Whole genome detection of rotavirus mixed infections in human, porcine and bovine samples co-infected with various rotavirus strains collected from sub-Saharan Africa. Infect. Genet. Evol..

[34] McDonald S.M., Matthijnssens J., McAllen J.K., Hine E., Overton L., Wang S., Lemey P., Zeller M., Van Ranst M., Spiro D.J. (2009). Evolutionary dynamics of human rotaviruses: Balancing reassortment with preferred genome constellations. PLoS Pathog..

[35] Koukou D.M., Michos A., Chatzichristou P., Trimis G., Tatsi E.B., Dellis C., Zachariadou L., Liakopoulou T., Chrousos G.P., Syriopoulou V. (2022). Rotavirus epidemiology and genotype distribution in hospitalised children, Greece, 2008 to 2020: A prospective multicentre study. Eurosurveillance.

[36] Kirkwood C.D. (2010). Genetic and antigenic diversity of human rotaviruses: Potential impact on vaccination programs. J. Infect. Dis..

[37] Agbemabiese C.A., Nakagomi T., Damanka S.A., Dennis F.E., Lartey B.L., Armah G.E., Nakagomi O. (2019). Sub-genotype phylogeny of the non-G, non-P genes of genotype 2 Rotavirus A strains. PLoS ONE.

[38] Bányai K., László B., Duque J., Steele A.D., Nelson E.A.S., Gentsch J.R., Parashar U.D. (2012). Systematic review of regional and temporal trends in global rotavirus strain diversity in the pre rotavirus vaccine era: Insights for understanding the impact of rotavirus vaccination programs. Vaccine.

[39] Jiang V., Jiang B., Tate J., Parashar U.D., Patel M.M. (2010). Performance of rotavirus vaccines in developed and developing countries. Hum. Vaccines.

[40] Steele A.D., Neuzil K.M., Cunliffe N.A., Madhi S.A., Bos P., Ngwira B., Witte D., Todd S., Louw C., Kirsten M. (2012). Human rotavirus vaccine Rotarix™ provides protection against diverse circulating rotavirus strains in African infants: A randomized controlled trial. BMC Infect. Dis..

[41] Hoshino Y., Honma S., Jones R.W., Ross J., Santos N., Gentsch J.R., Kapikian A.Z., Hesse R.A. (2005). A porcine G9 rotavirus strain shares neutralization and VP7 phylogenetic sequence lineage 3 characteristics with contemporary human G9 rotavirus strains. Virology.

[42] Page N., Esona M., Armah G., Nyangao J., Mwenda J., Sebunya T., Basu G., Pyndiah N., Potgieter N., Geyer A. (2010). Emergence and characterization of serotype G9 rotavirus strains from Africa. J. Infect. Dis..

[43] Santos N., Hoshino Y. (2005). Global distribution of rotavirus serotypes/genotypes and its implication for the development and implementation of an effective rotavirus vaccine. Rev. Med. Virol..

[44] Mullick S., Mandal P., Nayak M.K., Ghosh S., De P., Rajendran K., Bhattacharya M.K., Mitra U., Ramamurthy T., Kobayashi N. (2014). Hospital based surveillance and genetic characterization of rotavirus strains in children (<5 years) with acute gastroenteritis in Kolkata, India, revealed resurgence of G9 and G2 genotypes during 2011–2013. Vaccine.

[45] Jere K.C., Mlera L., O’Neill H.G., Potgieter A.C., Page N.A., Seheri M.L., van Dijk A.A. (2011). Whole genome analyses of African G2, G8, G9, and G12 rotavirus strains using sequence-independent amplification and 454® pyrosequencing. J. Med. Virol..

[46] Burnett E., Parashar U.D., Winn A., Tate J.E. (2022). Trends in rotavirus laboratory detections and internet search volume before and after rotavirus vaccine introduction and in the context of the coronavirus disease 2019 pandemic—United States, 2000–2021. J. Infect. Dis..

[47] Nakagomi T., Doan Y.H., Dove W., Ngwira B., Iturriza-Gomara M., Nakagomi O., Cunliffe N.A. (2013). G8 rotaviruses with conserved genotype constellations detected in Malawi over 10 years (1997–2007) display frequent gene reassortment among strains co-circulating in humans. J. Gen. Virol..

[48] Martinez-Laso J., Román A., Head J., Cervera I., Rodríguez M., Rodríguez-Avial I., Picazo J.J. (2009). Phylogeny of G9 rotavirus genotype: A possible explanation of its origin and evolution. J. Clin. Virol..

[49] Vetter V., Gardner R.C., Debrus S., Benninghoff B., Pereira P. (2022). Established and new rotavirus vaccines: A comprehensive review for healthcare professionals. Hum. Vaccines Immunother..

[50] Ansaldi F., Pastorino B., Valle L., Durando P., Sticchi L., Tucci P., Biasci P., Lai P., Gasparini R., Icardi G. (2007). Molecular characterization of a new variant of rotavirus P [8] G9 predominant in a sentinel-based survey in central Italy. J. Clin. Microbiol..

[51] Tatte V.S., Chitambar D.S.D. (2011). Intragenotypic diversity in the VP4 encoding genes of rotavirus strains circulating in adolescent and adult cases of acute gastroenteritis in Pune, Western India: 1993 to 1996 and 2004 to 2007. J. Gen. Mol. Virol..

[52] Trinh Q.D., Nguyen T.A., Phan T.G., Khamrin P., Yan H., Le Hoang P., Maneekarn N., Li Y., Yagyu F., Okitsu S. (2007). Sequence analysis of the VP7 gene of human rotavirus G1 isolated in Japan, China, Thailand, and Vietnam in the context of changing distribution of rotavirus G-types. J. Med. Virol..

[53] Zeller M., Donato C., Trovao N.S., Cowley D., Heylen E., Donker N.C., McAllen J.K., Akopov A., Kirkness E.F., Lemey P. (2015). Genome-wide evolutionary analyses of G1P [8] strains isolated before and after rotavirus vaccine introduction. Genome Biol. Evol..

