# Supplementary material for: Genomic Analysis of Rwandan G9P[8] Rotavirus Strains Pre- and Post-RotaTeq® Vaccine Reveals Significant Distinct Sub-Clustering in a Post-Vaccination Cohort
Source: Viruses. 2023 Nov 25;15(12):2321. doi: 10.3390/v15122321 (PMC10747556; doi:10.3390/v15122321)
Supplement: Supplementary file 1 [file viruses-15-02321-s001.zip › Supplementary Material S2.pdf]

Supplementary Material S2: Maximum likelihood trees generated in this G9P[8] study

NSP1

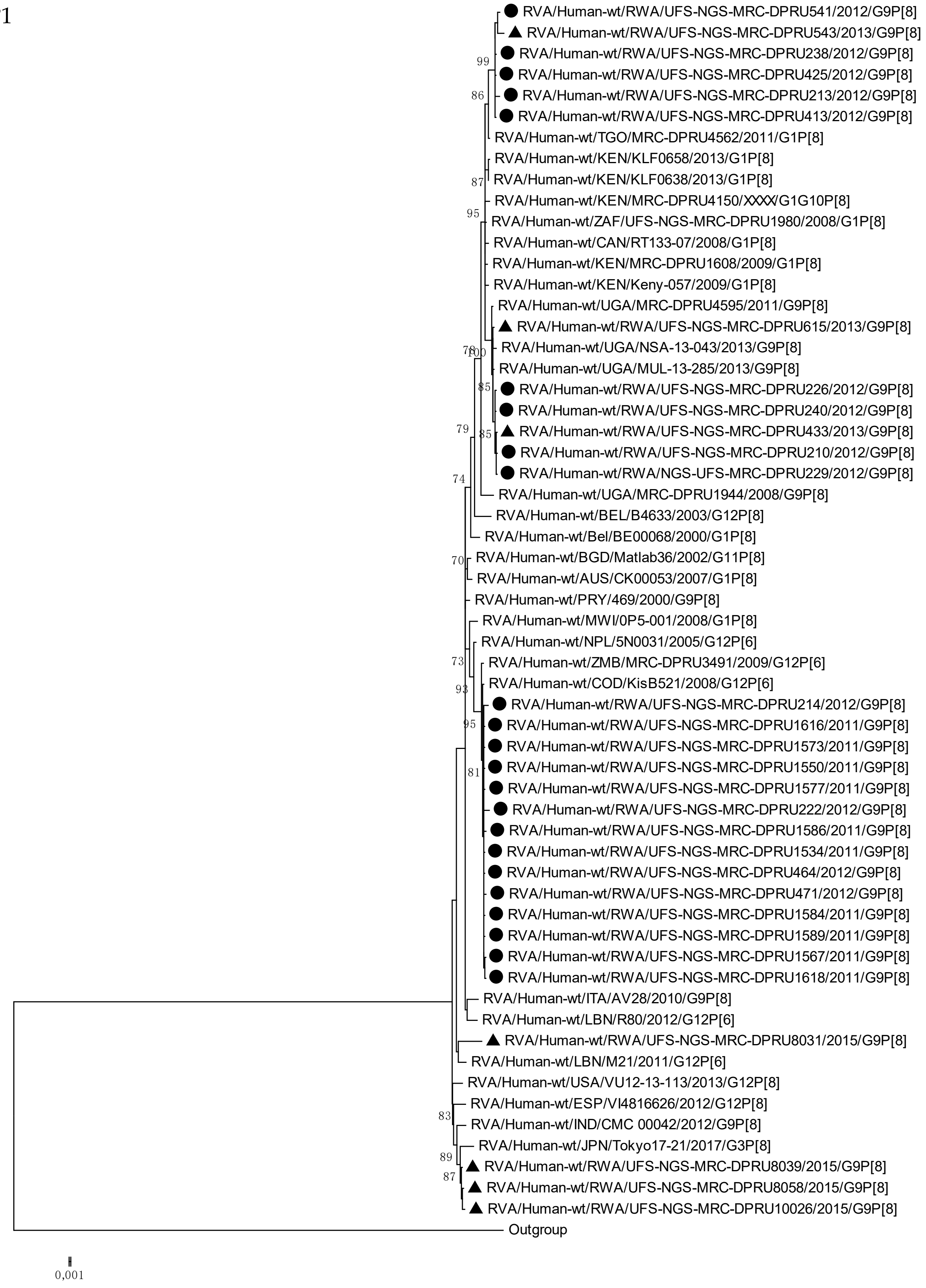

NSP2

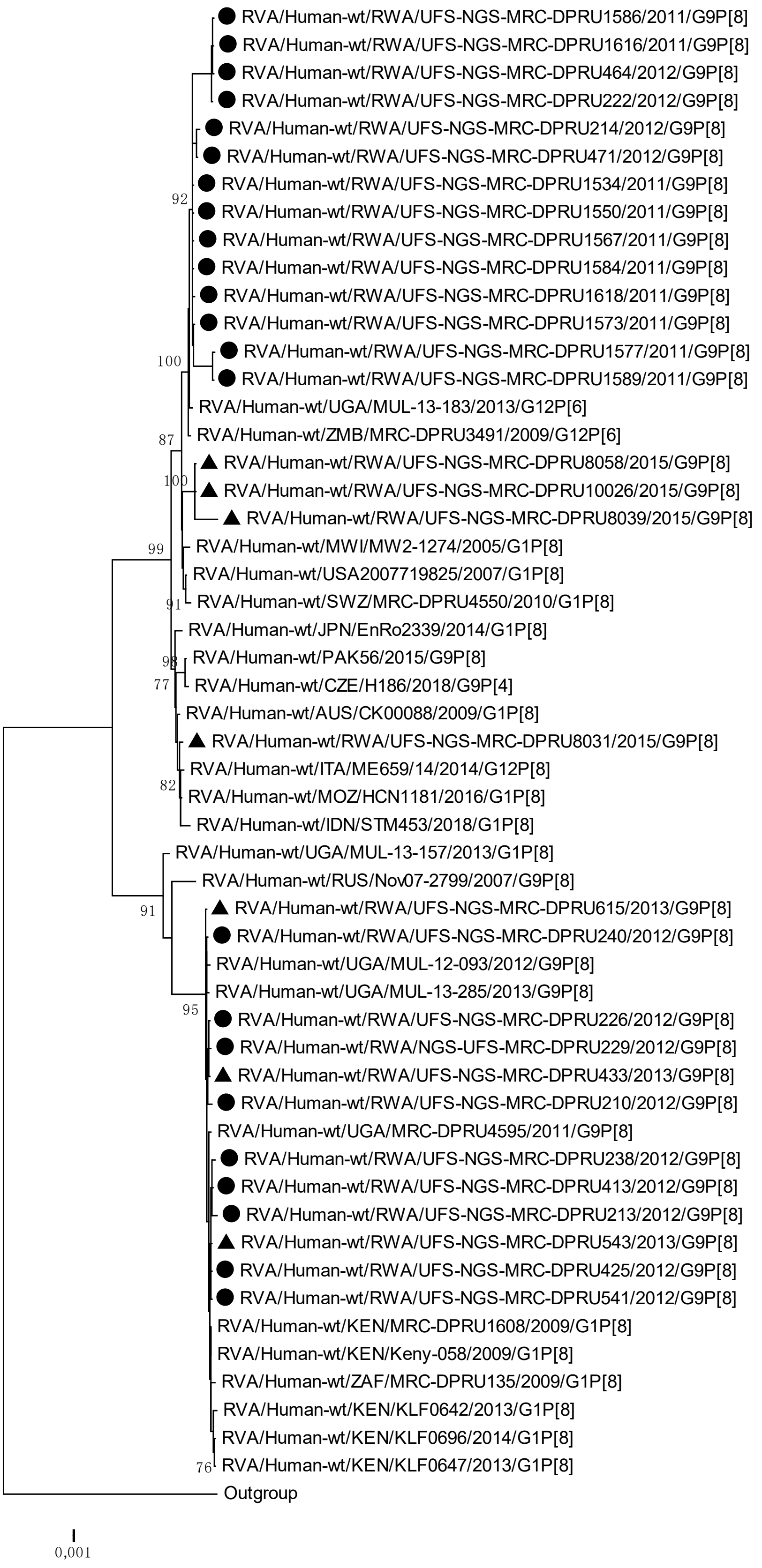
RVA/Human-wt/RWA/UFS-NGS-MRC-DPRU1586/2011/G9P[8]
RVA/Human-wt/RWA/UFS-NGS-MRC-DPRU1616/2011/G9P[8]
RVA/Human-wt/RWA/UFS-NGS-MRC-DPRU464/2012/G9P[8]
RVA/Human-wt/RWA/UFS-NGS-MRC-DPRU222/2012/G9P[8]
RVA/Human-wt/RWA/UFS-NGS-MRC-DPRU214/2012/G9P[8]
RVA/Human-wt/RWA/UFS-NGS-MRC-DPRU471/2012/G9P[8]
RVA/Human-wt/RWA/UFS-NGS-MRC-DPRU1534/2011/G9P[8]
92
RVA/Human-wt/RWA/UFS-NGS-MRC-DPRU1550/2011/G9P[8]
RVA/Human-wt/RWA/UFS-NGS-MRC-DPRU1567/2011/G9P[8]
RVA/Human-wt/RWA/UFS-NGS-MRC-DPRU1584/2011/G9P[8]
RVA/Human-wt/RWA/UFS-NGS-MRC-DPRU1618/2011/G9P[8]
RVA/Human-wt/RWA/UFS-NGS-MRC-DPRU1573/2011/G9P[8]
RVA/Human-wt/RWA/UFS-NGS-MRC-DPRU1577/2011/G9P[8]
100
RVA/Human-wt/RWA/UFS-NGS-MRC-DPRU1589/2011/G9P[8]
RVA/Human-wt/UGA/MUL-13-183/2013/G12P[6]
87
RVA/Human-wt/ZMB/MRC-DPRU3491/2009/G12P[6]
RVA/Human-wt/RWA/UFS-NGS-MRC-DPRU8058/2015/G9P[8]
100
RVA/Human-wt/RWA/UFS-NGS-MRC-DPRU10026/2015/G9P[8]
RVA/Human-wt/RWA/UFS-NGS-MRC-DPRU8039/2015/G9P[8]
RVA/Human-wt/MWI/MW2-1274/2005/G1P[8]
99
RVA/Human-wt/USA2007719825/2007/G1P[8]
RVA/Human-wt/SWZ/MRC-DPRU4550/2010/G1P[8]
91
RVA/Human-wt/JPN/EnRo2339/2014/G1P[8]
98
RVA/Human-wt/PAK56/2015/G9P[8]
77
RVA/Human-wt/CZE/H186/2018/G9P[4]
RVA/Human-wt/AUS/CK00088/2009/G1P[8]
RVA/Human-wt/RWA/UFS-NGS-MRC-DPRU8031/2015/G9P[8]
RVA/Human-wt/ITA/ME659/14/2014/G12P[8]
82
RVA/Human-wt/MOZ/HCN1181/2016/G1P[8]
RVA/Human-wt/IDN/STM453/2018/G1P[8]
RVA/Human-wt/UGA/MUL-13-157/2013/G1P[8]
RVA/Human-wt/RUS/Nov07-2799/2007/G9P[8]
91
RVA/Human-wt/RWA/UFS-NGS-MRC-DPRU615/2013/G9P[8]
RVA/Human-wt/RWA/UFS-NGS-MRC-DPRU240/2012/G9P[8]
RVA/Human-wt/UGA/MUL-12-093/2012/G9P[8]
RVA/Human-wt/UGA/MUL-13-285/2013/G9P[8]
95
RVA/Human-wt/RWA/UFS-NGS-MRC-DPRU226/2012/G9P[8]
RVA/Human-wt/RWA/NGS-UFS-MRC-DPRU229/2012/G9P[8]
RVA/Human-wt/RWA/UFS-NGS-MRC-DPRU433/2013/G9P[8]
RVA/Human-wt/RWA/UFS-NGS-MRC-DPRU210/2012/G9P[8]
RVA/Human-wt/UGA/MRC-DPRU4595/2011/G9P[8]
RVA/Human-wt/RWA/UFS-NGS-MRC-DPRU238/2012/G9P[8]
RVA/Human-wt/RWA/UFS-NGS-MRC-DPRU413/2012/G9P[8]
RVA/Human-wt/RWA/UFS-NGS-MRC-DPRU213/2012/G9P[8]
RVA/Human-wt/RWA/UFS-NGS-MRC-DPRU543/2013/G9P[8]
RVA/Human-wt/RWA/UFS-NGS-MRC-DPRU425/2012/G9P[8]
RVA/Human-wt/RWA/UFS-NGS-MRC-DPRU541/2012/G9P[8]
RVA/Human-wt/KEN/MRC-DPRU1608/2009/G1P[8]
RVA/Human-wt/KEN/Keny-058/2009/G1P[8]
RVA/Human-wt/ZAF/MRC-DPRU135/2009/G1P[8]
RVA/Human-wt/KEN/KLF0642/2013/G1P[8]
RVA/Human-wt/KEN/KLF0696/2014/G1P[8]
76
RVA/Human-wt/KEN/KLF0647/2013/G1P[8]
Outgroup
0,001

NSP3

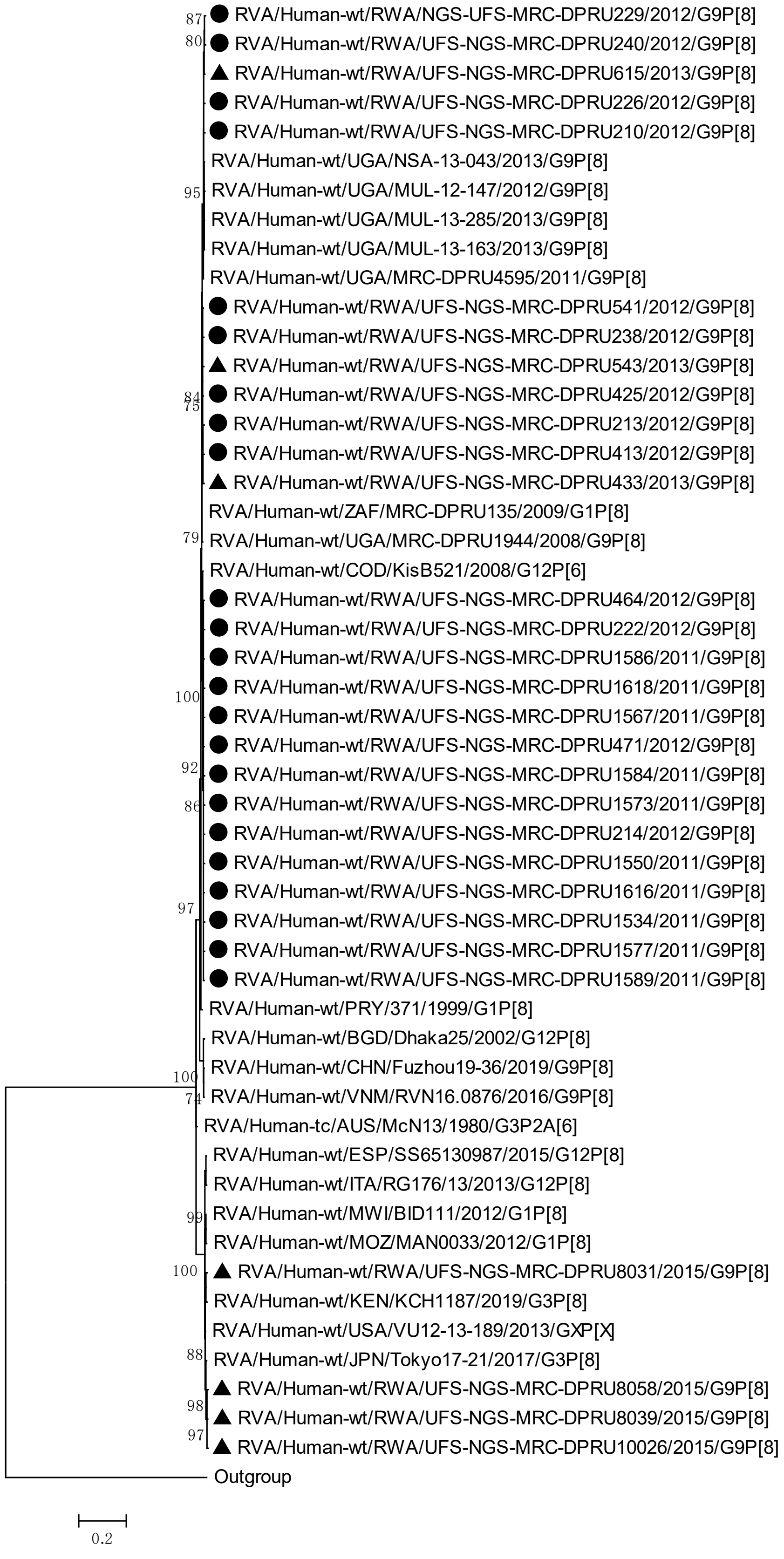
RVA/Human-wt/RWA/NGS-UFS-MRC-DPRU229/2012/G9P[8]
RVA/Human-wt/RWA/UFS-NGS-MRC-DPRU240/2012/G9P[8]
RVA/Human-wt/RWA/UFS-NGS-MRC-DPRU615/2013/G9P[8]
RVA/Human-wt/RWA/UFS-NGS-MRC-DPRU226/2012/G9P[8]
RVA/Human-wt/RWA/UFS-NGS-MRC-DPRU210/2012/G9P[8]
RVA/Human-wt/UGA/NSA-13-043/2013/G9P[8]
RVA/Human-wt/UGA/MUL-12-147/2012/G9P[8]
RVA/Human-wt/UGA/MUL-13-285/2013/G9P[8]
RVA/Human-wt/UGA/MUL-13-163/2013/G9P[8]
RVA/Human-wt/UGA/MRC-DPRU4595/2011/G9P[8]
RVA/Human-wt/RWA/UFS-NGS-MRC-DPRU541/2012/G9P[8]
RVA/Human-wt/RWA/UFS-NGS-MRC-DPRU238/2012/G9P[8]
RVA/Human-wt/RWA/UFS-NGS-MRC-DPRU543/2013/G9P[8]
RVA/Human-wt/RWA/UFS-NGS-MRC-DPRU425/2012/G9P[8]
RVA/Human-wt/RWA/UFS-NGS-MRC-DPRU213/2012/G9P[8]
RVA/Human-wt/RWA/UFS-NGS-MRC-DPRU413/2012/G9P[8]
RVA/Human-wt/RWA/UFS-NGS-MRC-DPRU433/2013/G9P[8]
RVA/Human-wt/ZAF/MRC-DPRU135/2009/G1P[8]
RVA/Human-wt/UGA/MRC-DPRU1944/2008/G9P[8]
RVA/Human-wt/COD/KisB521/2008/G12P[6]
RVA/Human-wt/RWA/UFS-NGS-MRC-DPRU464/2012/G9P[8]
RVA/Human-wt/RWA/UFS-NGS-MRC-DPRU222/2012/G9P[8]
RVA/Human-wt/RWA/UFS-NGS-MRC-DPRU1586/2011/G9P[8]
RVA/Human-wt/RWA/UFS-NGS-MRC-DPRU1618/2011/G9P[8]
RVA/Human-wt/RWA/UFS-NGS-MRC-DPRU1567/2011/G9P[8]
RVA/Human-wt/RWA/UFS-NGS-MRC-DPRU471/2012/G9P[8]
RVA/Human-wt/RWA/UFS-NGS-MRC-DPRU1584/2011/G9P[8]
RVA/Human-wt/RWA/UFS-NGS-MRC-DPRU1573/2011/G9P[8]
RVA/Human-wt/RWA/UFS-NGS-MRC-DPRU214/2012/G9P[8]
RVA/Human-wt/RWA/UFS-NGS-MRC-DPRU1550/2011/G9P[8]
RVA/Human-wt/RWA/UFS-NGS-MRC-DPRU1616/2011/G9P[8]
RVA/Human-wt/RWA/UFS-NGS-MRC-DPRU1534/2011/G9P[8]
RVA/Human-wt/RWA/UFS-NGS-MRC-DPRU1577/2011/G9P[8]
RVA/Human-wt/RWA/UFS-NGS-MRC-DPRU1589/2011/G9P[8]
RVA/Human-wt/PRY/371/1999/G1P[8]
RVA/Human-wt/BGD/Dhaka25/2002/G12P[8]
RVA/Human-wt/CHN/Fuzhou19-36/2019/G9P[8]
RVA/Human-wt/VNM/RVN16.0876/2016/G9P[8]
RVA/Human-tc/AUS/McN13/1980/G3P2A[6]
RVA/Human-wt/ESP/SS65130987/2015/G12P[8]
RVA/Human-wt/ITA/RG176/13/2013/G12P[8]
RVA/Human-wt/MWI/BID111/2012/G1P[8]
RVA/Human-wt/MOZ/MAN0033/2012/G1P[8]
RVA/Human-wt/RWA/UFS-NGS-MRC-DPRU8031/2015/G9P[8]
RVA/Human-wt/KEN/KCH1187/2019/G3P[8]
RVA/Human-wt/USA/VU12-13-189/2013/GXP[X]
RVA/Human-wt/JPN/Tokyo17-21/2017/G3P[8]
RVA/Human-wt/RWA/UFS-NGS-MRC-DPRU8058/2015/G9P[8]
RVA/Human-wt/RWA/UFS-NGS-MRC-DPRU8039/2015/G9P[8]
RVA/Human-wt/RWA/UFS-NGS-MRC-DPRU10026/2015/G9P[8]
Outgroup
0.2

NSP4

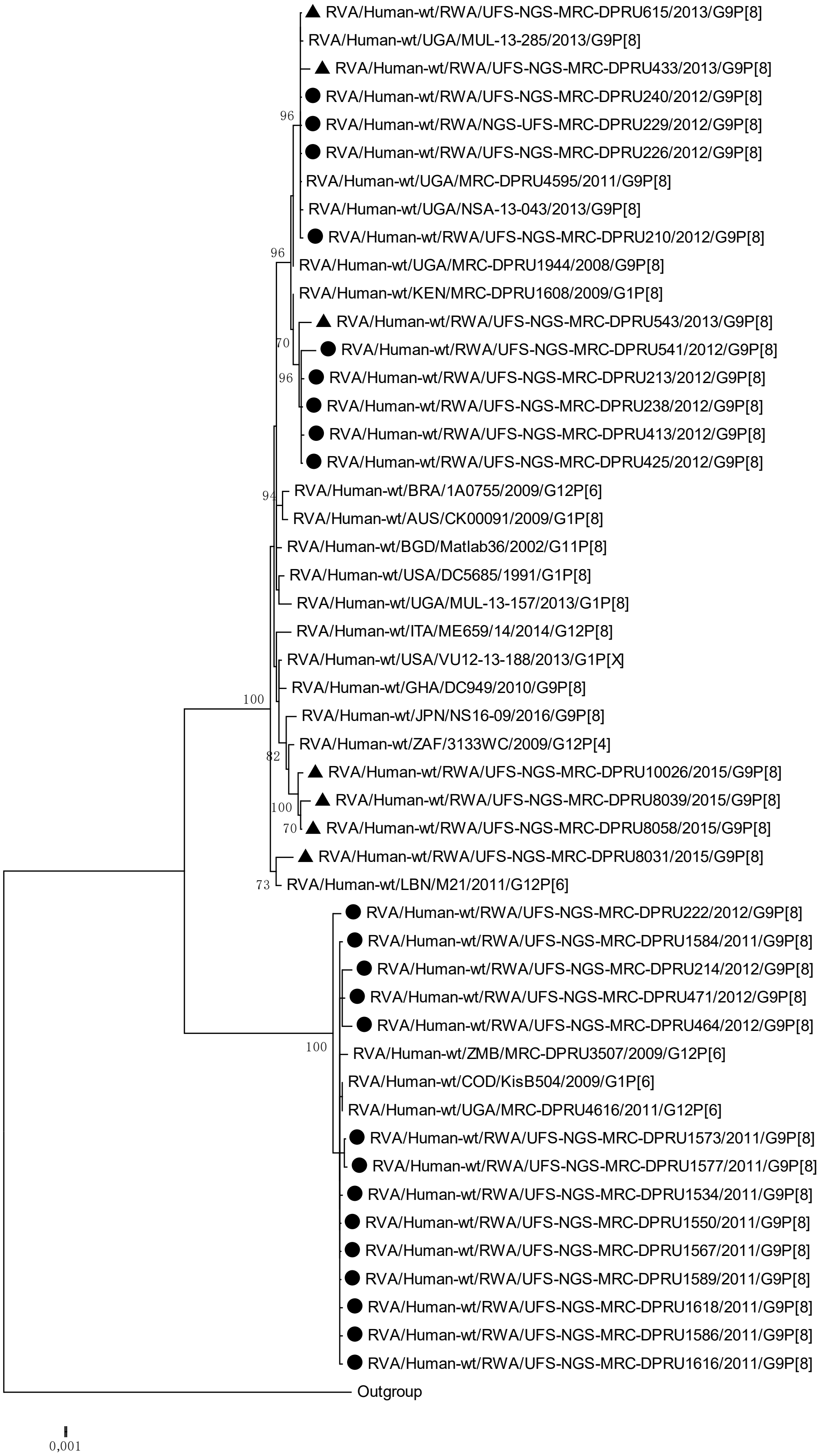
RVA/Human-wt/RWA/UFS-NGS-MRC-DPRU615/2013/G9P[8]
RVA/Human-wt/UGA/MUL-13-285/2013/G9P[8]
RVA/Human-wt/RWA/UFS-NGS-MRC-DPRU433/2013/G9P[8]
RVA/Human-wt/RWA/UFS-NGS-MRC-DPRU240/2012/G9P[8]
RVA/Human-wt/RWA/NGS-UFS-MRC-DPRU229/2012/G9P[8]
RVA/Human-wt/RWA/UFS-NGS-MRC-DPRU226/2012/G9P[8]
RVA/Human-wt/UGA/MRC-DPRU4595/2011/G9P[8]
RVA/Human-wt/UGA/NSA-13-043/2013/G9P[8]
RVA/Human-wt/RWA/UFS-NGS-MRC-DPRU210/2012/G9P[8]
RVA/Human-wt/UGA/MRC-DPRU1944/2008/G9P[8]
RVA/Human-wt/KEN/MRC-DPRU1608/2009/G1P[8]
RVA/Human-wt/RWA/UFS-NGS-MRC-DPRU543/2013/G9P[8]
RVA/Human-wt/RWA/UFS-NGS-MRC-DPRU541/2012/G9P[8]
RVA/Human-wt/RWA/UFS-NGS-MRC-DPRU213/2012/G9P[8]
RVA/Human-wt/RWA/UFS-NGS-MRC-DPRU238/2012/G9P[8]
RVA/Human-wt/RWA/UFS-NGS-MRC-DPRU413/2012/G9P[8]
RVA/Human-wt/RWA/UFS-NGS-MRC-DPRU425/2012/G9P[8]
RVA/Human-wt/BRA/1A0755/2009/G12P[6]
RVA/Human-wt/AUS/CK00091/2009/G1P[8]
RVA/Human-wt/BGD/Matlab36/2002/G11P[8]
RVA/Human-wt/USA/DC5685/1991/G1P[8]
RVA/Human-wt/UGA/MUL-13-157/2013/G1P[8]
RVA/Human-wt/ITA/ME659/14/2014/G12P[8]
RVA/Human-wt/USA/VU12-13-188/2013/G1P[X]
RVA/Human-wt/GHA/DC949/2010/G9P[8]
RVA/Human-wt/JPN/NS16-09/2016/G9P[8]
RVA/Human-wt/ZAF/3133WC/2009/G12P[4]
RVA/Human-wt/RWA/UFS-NGS-MRC-DPRU10026/2015/G9P[8]
RVA/Human-wt/RWA/UFS-NGS-MRC-DPRU8039/2015/G9P[8]
RVA/Human-wt/RWA/UFS-NGS-MRC-DPRU8058/2015/G9P[8]
RVA/Human-wt/RWA/UFS-NGS-MRC-DPRU8031/2015/G9P[8]
RVA/Human-wt/LBN/M21/2011/G12P[6]
RVA/Human-wt/RWA/UFS-NGS-MRC-DPRU222/2012/G9P[8]
RVA/Human-wt/RWA/UFS-NGS-MRC-DPRU1584/2011/G9P[8]
RVA/Human-wt/RWA/UFS-NGS-MRC-DPRU214/2012/G9P[8]
RVA/Human-wt/RWA/UFS-NGS-MRC-DPRU471/2012/G9P[8]
RVA/Human-wt/RWA/UFS-NGS-MRC-DPRU464/2012/G9P[8]
RVA/Human-wt/ZMB/MRC-DPRU3507/2009/G12P[6]
RVA/Human-wt/COD/KisB504/2009/G1P[6]
RVA/Human-wt/UGA/MRC-DPRU4616/2011/G12P[6]
RVA/Human-wt/RWA/UFS-NGS-MRC-DPRU1573/2011/G9P[8]
RVA/Human-wt/RWA/UFS-NGS-MRC-DPRU1577/2011/G9P[8]
RVA/Human-wt/RWA/UFS-NGS-MRC-DPRU1534/2011/G9P[8]
RVA/Human-wt/RWA/UFS-NGS-MRC-DPRU1550/2011/G9P[8]
RVA/Human-wt/RWA/UFS-NGS-MRC-DPRU1567/2011/G9P[8]
RVA/Human-wt/RWA/UFS-NGS-MRC-DPRU1589/2011/G9P[8]
RVA/Human-wt/RWA/UFS-NGS-MRC-DPRU1618/2011/G9P[8]
RVA/Human-wt/RWA/UFS-NGS-MRC-DPRU1586/2011/G9P[8]
RVA/Human-wt/RWA/UFS-NGS-MRC-DPRU1616/2011/G9P[8]
Outgroup
96
96
70
96
94
100
82
100
70
73
100
0,001

NSP5

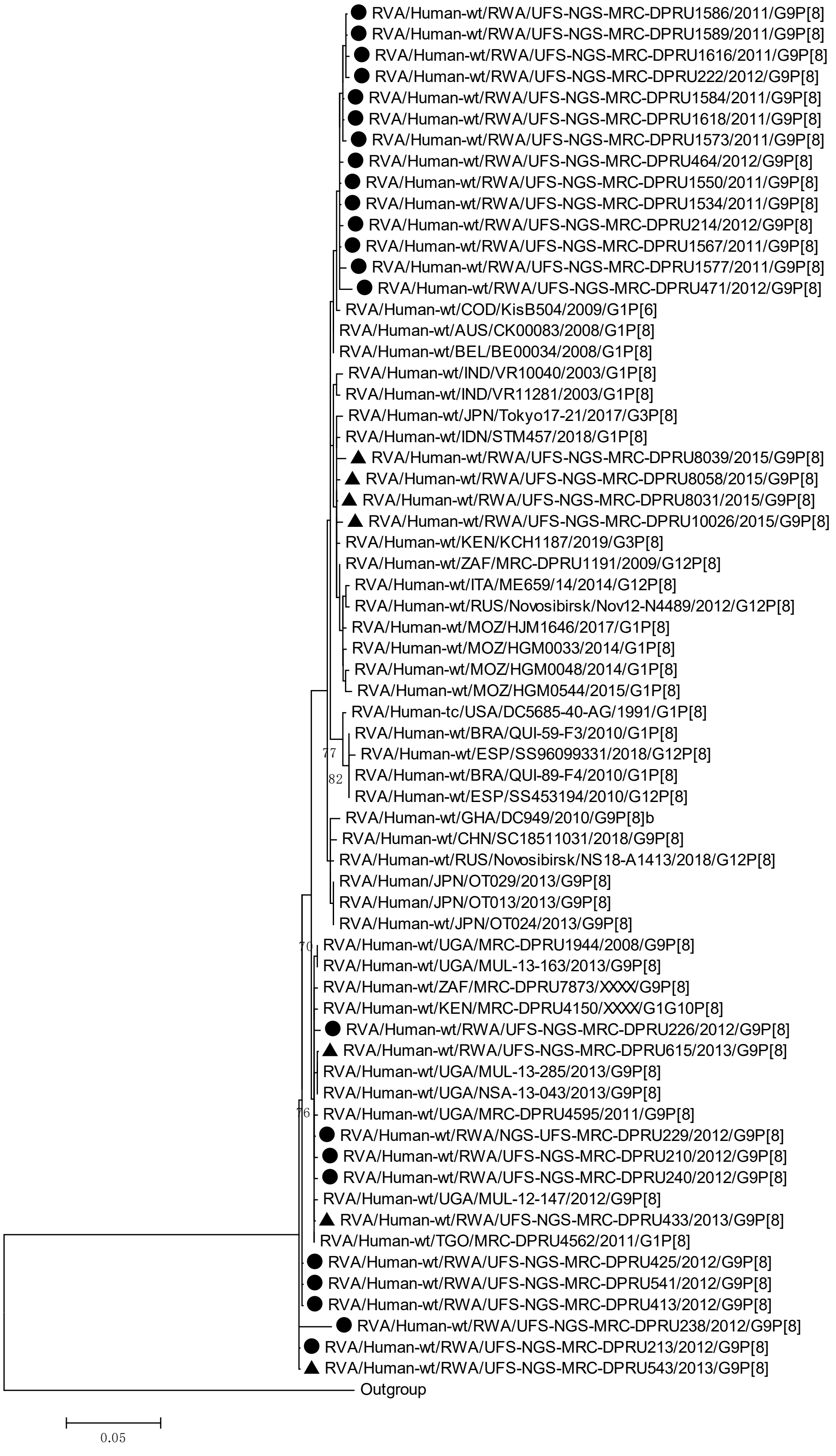
RVA/Human-wt/RWA/UFS-NGS-MRC-DPRU1586/2011/G9P[8]
RVA/Human-wt/RWA/UFS-NGS-MRC-DPRU1589/2011/G9P[8]
RVA/Human-wt/RWA/UFS-NGS-MRC-DPRU1616/2011/G9P[8]
RVA/Human-wt/RWA/UFS-NGS-MRC-DPRU222/2012/G9P[8]
RVA/Human-wt/RWA/UFS-NGS-MRC-DPRU1584/2011/G9P[8]
RVA/Human-wt/RWA/UFS-NGS-MRC-DPRU1618/2011/G9P[8]
RVA/Human-wt/RWA/UFS-NGS-MRC-DPRU1573/2011/G9P[8]
RVA/Human-wt/RWA/UFS-NGS-MRC-DPRU464/2012/G9P[8]
RVA/Human-wt/RWA/UFS-NGS-MRC-DPRU1550/2011/G9P[8]
RVA/Human-wt/RWA/UFS-NGS-MRC-DPRU1534/2011/G9P[8]
RVA/Human-wt/RWA/UFS-NGS-MRC-DPRU214/2012/G9P[8]
RVA/Human-wt/RWA/UFS-NGS-MRC-DPRU1567/2011/G9P[8]
RVA/Human-wt/RWA/UFS-NGS-MRC-DPRU1577/2011/G9P[8]
RVA/Human-wt/RWA/UFS-NGS-MRC-DPRU471/2012/G9P[8]
RVA/Human-wt/COD/KisB504/2009/G1P[6]
RVA/Human-wt/AUS/CK00083/2008/G1P[8]
RVA/Human-wt/BEL/BE00034/2008/G1P[8]
RVA/Human-wt/IND/VR10040/2003/G1P[8]
RVA/Human-wt/IND/VR11281/2003/G1P[8]
RVA/Human-wt/JPN/Tokyo17-21/2017/G3P[8]
RVA/Human-wt/IDN/STM457/2018/G1P[8]
RVA/Human-wt/RWA/UFS-NGS-MRC-DPRU8039/2015/G9P[8]
RVA/Human-wt/RWA/UFS-NGS-MRC-DPRU8058/2015/G9P[8]
RVA/Human-wt/RWA/UFS-NGS-MRC-DPRU8031/2015/G9P[8]
RVA/Human-wt/RWA/UFS-NGS-MRC-DPRU10026/2015/G9P[8]
RVA/Human-wt/KEN/KCH1187/2019/G3P[8]
RVA/Human-wt/ZAF/MRC-DPRU1191/2009/G12P[8]
RVA/Human-wt/ITA/ME659/14/2014/G12P[8]
RVA/Human-wt/RUS/Novosibirsk/Nov12-N4489/2012/G12P[8]
RVA/Human-wt/MOZ/HJM1646/2017/G1P[8]
RVA/Human-wt/MOZ/HGM0033/2014/G1P[8]
RVA/Human-wt/MOZ/HGM0048/2014/G1P[8]
RVA/Human-wt/MOZ/HGM0544/2015/G1P[8]
RVA/Human-tc/USA/DC5685-40-AG/1991/G1P[8]
RVA/Human-wt/BRA/QUI-59-F3/2010/G1P[8]
77
RVA/Human-wt/ESP/SS96099331/2018/G12P[8]
82
RVA/Human-wt/BRA/QUI-89-F4/2010/G1P[8]
RVA/Human-wt/ESP/SS453194/2010/G12P[8]
RVA/Human-wt/GHA/DC949/2010/G9P[8]b
RVA/Human-wt/CHN/SC18511031/2018/G9P[8]
RVA/Human-wt/RUS/Novosibirsk/NS18-A1413/2018/G12P[8]
RVA/Human/JPN/OT029/2013/G9P[8]
RVA/Human/JPN/OT013/2013/G9P[8]
RVA/Human-wt/JPN/OT024/2013/G9P[8]
70
RVA/Human-wt/UGA/MRC-DPRU1944/2008/G9P[8]
RVA/Human-wt/UGA/MUL-13-163/2013/G9P[8]
RVA/Human-wt/ZAF/MRC-DPRU7873/XXXX/G9P[8]
RVA/Human-wt/KEN/MRC-DPRU4150/XXXX/G1G10P[8]
RVA/Human-wt/RWA/UFS-NGS-MRC-DPRU226/2012/G9P[8]
RVA/Human-wt/RWA/UFS-NGS-MRC-DPRU615/2013/G9P[8]
RVA/Human-wt/UGA/MUL-13-285/2013/G9P[8]
RVA/Human-wt/UGA/NSA-13-043/2013/G9P[8]
76
RVA/Human-wt/UGA/MRC-DPRU4595/2011/G9P[8]
RVA/Human-wt/RWA/NGS-UFS-MRC-DPRU229/2012/G9P[8]
RVA/Human-wt/RWA/UFS-NGS-MRC-DPRU210/2012/G9P[8]
RVA/Human-wt/RWA/UFS-NGS-MRC-DPRU240/2012/G9P[8]
RVA/Human-wt/UGA/MUL-12-147/2012/G9P[8]
RVA/Human-wt/RWA/UFS-NGS-MRC-DPRU433/2013/G9P[8]
RVA/Human-wt/TGO/MRC-DPRU4562/2011/G1P[8]
RVA/Human-wt/RWA/UFS-NGS-MRC-DPRU425/2012/G9P[8]
RVA/Human-wt/RWA/UFS-NGS-MRC-DPRU541/2012/G9P[8]
RVA/Human-wt/RWA/UFS-NGS-MRC-DPRU413/2012/G9P[8]
RVA/Human-wt/RWA/UFS-NGS-MRC-DPRU238/2012/G9P[8]
RVA/Human-wt/RWA/UFS-NGS-MRC-DPRU213/2012/G9P[8]
RVA/Human-wt/RWA/UFS-NGS-MRC-DPRU543/2013/G9P[8]
Outgroup
0.05

VP1

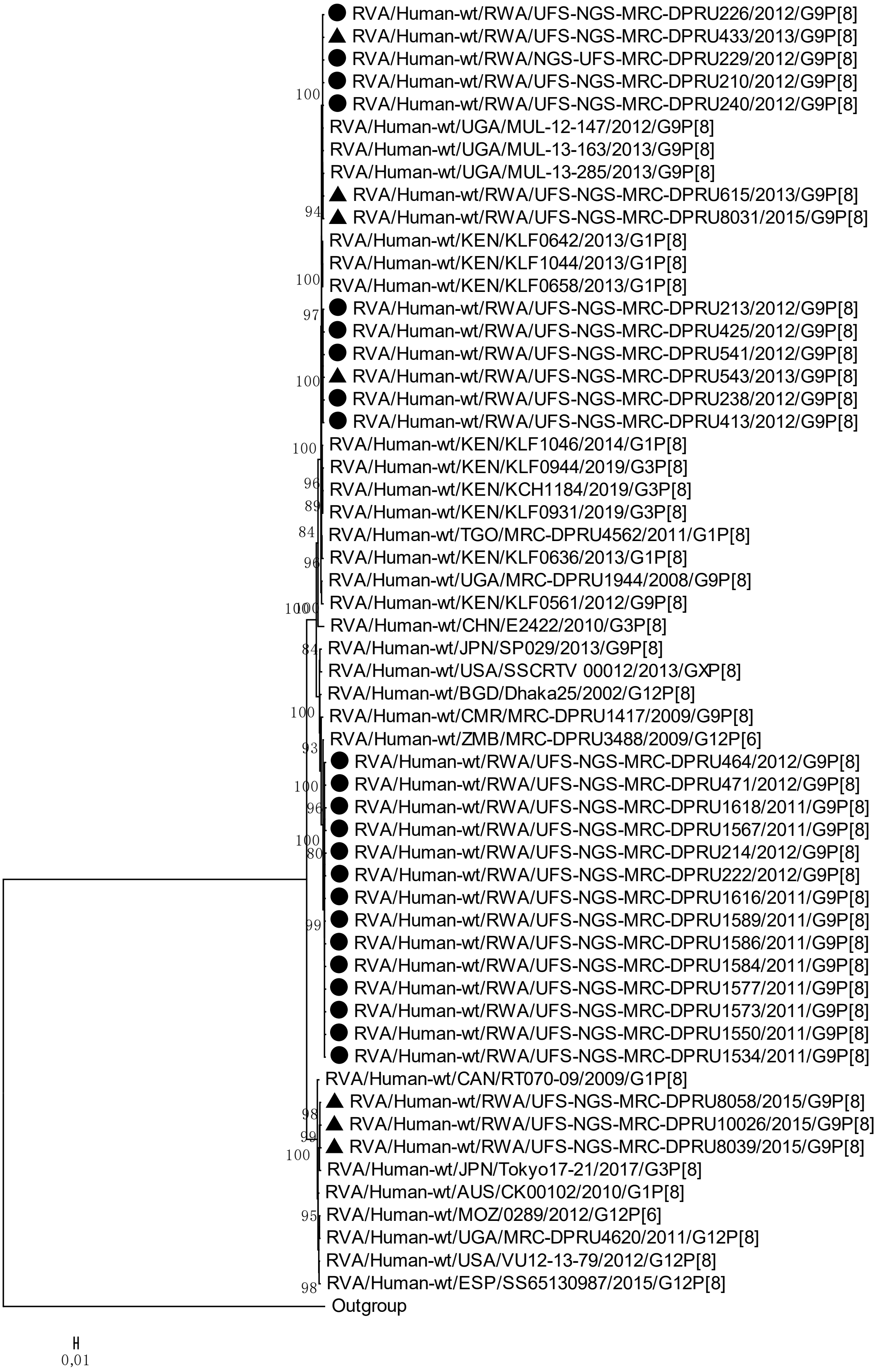
RVA/Human-wt/RWA/UFS-NGS-MRC-DPRU226/2012/G9P[8]
RVA/Human-wt/RWA/UFS-NGS-MRC-DPRU433/2013/G9P[8]
RVA/Human-wt/RWA/NGS-UFS-MRC-DPRU229/2012/G9P[8]
RVA/Human-wt/RWA/UFS-NGS-MRC-DPRU210/2012/G9P[8]
RVA/Human-wt/RWA/UFS-NGS-MRC-DPRU240/2012/G9P[8]
RVA/Human-wt/UGA/MUL-12-147/2012/G9P[8]
RVA/Human-wt/UGA/MUL-13-163/2013/G9P[8]
RVA/Human-wt/UGA/MUL-13-285/2013/G9P[8]
RVA/Human-wt/RWA/UFS-NGS-MRC-DPRU615/2013/G9P[8]
RVA/Human-wt/RWA/UFS-NGS-MRC-DPRU8031/2015/G9P[8]
RVA/Human-wt/KEN/KLF0642/2013/G1P[8]
RVA/Human-wt/KEN/KLF1044/2013/G1P[8]
RVA/Human-wt/KEN/KLF0658/2013/G1P[8]
RVA/Human-wt/RWA/UFS-NGS-MRC-DPRU213/2012/G9P[8]
RVA/Human-wt/RWA/UFS-NGS-MRC-DPRU425/2012/G9P[8]
RVA/Human-wt/RWA/UFS-NGS-MRC-DPRU541/2012/G9P[8]
RVA/Human-wt/RWA/UFS-NGS-MRC-DPRU543/2013/G9P[8]
RVA/Human-wt/RWA/UFS-NGS-MRC-DPRU238/2012/G9P[8]
RVA/Human-wt/RWA/UFS-NGS-MRC-DPRU413/2012/G9P[8]
RVA/Human-wt/KEN/KLF1046/2014/G1P[8]
RVA/Human-wt/KEN/KLF0944/2019/G3P[8]
RVA/Human-wt/KEN/KCH1184/2019/G3P[8]
RVA/Human-wt/KEN/KLF0931/2019/G3P[8]
RVA/Human-wt/TGO/MRC-DPRU4562/2011/G1P[8]
RVA/Human-wt/KEN/KLF0636/2013/G1P[8]
RVA/Human-wt/UGA/MRC-DPRU1944/2008/G9P[8]
RVA/Human-wt/KEN/KLF0561/2012/G9P[8]
RVA/Human-wt/CHN/E2422/2010/G3P[8]
RVA/Human-wt/JPN/SP029/2013/G9P[8]
RVA/Human-wt/USA/SSCRTV 00012/2013/GXP[8]
RVA/Human-wt/BGD/Dhaka25/2002/G12P[8]
RVA/Human-wt/CMR/MRC-DPRU1417/2009/G9P[8]
RVA/Human-wt/ZMB/MRC-DPRU3488/2009/G12P[6]
RVA/Human-wt/RWA/UFS-NGS-MRC-DPRU464/2012/G9P[8]
RVA/Human-wt/RWA/UFS-NGS-MRC-DPRU471/2012/G9P[8]
RVA/Human-wt/RWA/UFS-NGS-MRC-DPRU1618/2011/G9P[8]
RVA/Human-wt/RWA/UFS-NGS-MRC-DPRU1567/2011/G9P[8]
RVA/Human-wt/RWA/UFS-NGS-MRC-DPRU214/2012/G9P[8]
RVA/Human-wt/RWA/UFS-NGS-MRC-DPRU222/2012/G9P[8]
RVA/Human-wt/RWA/UFS-NGS-MRC-DPRU1616/2011/G9P[8]
RVA/Human-wt/RWA/UFS-NGS-MRC-DPRU1589/2011/G9P[8]
RVA/Human-wt/RWA/UFS-NGS-MRC-DPRU1586/2011/G9P[8]
RVA/Human-wt/RWA/UFS-NGS-MRC-DPRU1584/2011/G9P[8]
RVA/Human-wt/RWA/UFS-NGS-MRC-DPRU1577/2011/G9P[8]
RVA/Human-wt/RWA/UFS-NGS-MRC-DPRU1573/2011/G9P[8]
RVA/Human-wt/RWA/UFS-NGS-MRC-DPRU1550/2011/G9P[8]
RVA/Human-wt/RWA/UFS-NGS-MRC-DPRU1534/2011/G9P[8]
RVA/Human-wt/CAN/RT070-09/2009/G1P[8]
RVA/Human-wt/RWA/UFS-NGS-MRC-DPRU8058/2015/G9P[8]
RVA/Human-wt/RWA/UFS-NGS-MRC-DPRU10026/2015/G9P[8]
RVA/Human-wt/RWA/UFS-NGS-MRC-DPRU8039/2015/G9P[8]
RVA/Human-wt/JPN/Tokyo17-21/2017/G3P[8]
RVA/Human-wt/AUS/CK00102/2010/G1P[8]
RVA/Human-wt/MOZ/0289/2012/G12P[6]
RVA/Human-wt/UGA/MRC-DPRU4620/2011/G12P[8]
RVA/Human-wt/USA/VU12-13-79/2012/G12P[8]
RVA/Human-wt/ESP/SS65130987/2015/G12P[8]
Outgroup
0,01

VP2

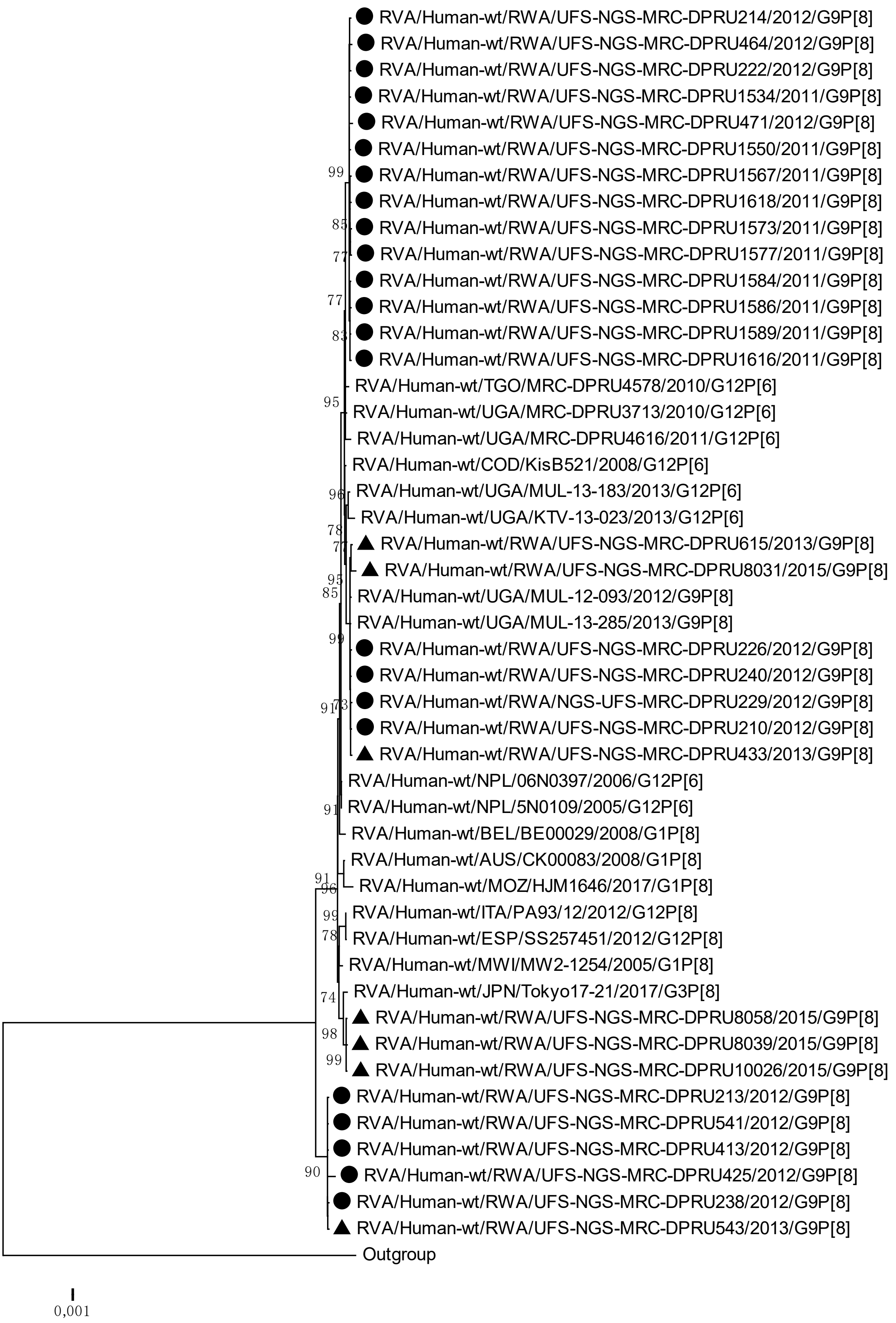
RVA/Human-wt/RWA/UFS-NGS-MRC-DPRU214/2012/G9P[8]
RVA/Human-wt/RWA/UFS-NGS-MRC-DPRU464/2012/G9P[8]
RVA/Human-wt/RWA/UFS-NGS-MRC-DPRU222/2012/G9P[8]
RVA/Human-wt/RWA/UFS-NGS-MRC-DPRU1534/2011/G9P[8]
RVA/Human-wt/RWA/UFS-NGS-MRC-DPRU471/2012/G9P[8]
RVA/Human-wt/RWA/UFS-NGS-MRC-DPRU1550/2011/G9P[8]
RVA/Human-wt/RWA/UFS-NGS-MRC-DPRU1567/2011/G9P[8]
RVA/Human-wt/RWA/UFS-NGS-MRC-DPRU1618/2011/G9P[8]
RVA/Human-wt/RWA/UFS-NGS-MRC-DPRU1573/2011/G9P[8]
RVA/Human-wt/RWA/UFS-NGS-MRC-DPRU1577/2011/G9P[8]
RVA/Human-wt/RWA/UFS-NGS-MRC-DPRU1584/2011/G9P[8]
RVA/Human-wt/RWA/UFS-NGS-MRC-DPRU1586/2011/G9P[8]
RVA/Human-wt/RWA/UFS-NGS-MRC-DPRU1589/2011/G9P[8]
RVA/Human-wt/RWA/UFS-NGS-MRC-DPRU1616/2011/G9P[8]
RVA/Human-wt/TGO/MRC-DPRU4578/2010/G12P[6]
RVA/Human-wt/UGA/MRC-DPRU3713/2010/G12P[6]
RVA/Human-wt/UGA/MRC-DPRU4616/2011/G12P[6]
RVA/Human-wt/COD/KisB521/2008/G12P[6]
RVA/Human-wt/UGA/MUL-13-183/2013/G12P[6]
RVA/Human-wt/UGA/KTV-13-023/2013/G12P[6]
RVA/Human-wt/RWA/UFS-NGS-MRC-DPRU615/2013/G9P[8]
RVA/Human-wt/RWA/UFS-NGS-MRC-DPRU8031/2015/G9P[8]
RVA/Human-wt/UGA/MUL-12-093/2012/G9P[8]
RVA/Human-wt/UGA/MUL-13-285/2013/G9P[8]
RVA/Human-wt/RWA/UFS-NGS-MRC-DPRU226/2012/G9P[8]
RVA/Human-wt/RWA/UFS-NGS-MRC-DPRU240/2012/G9P[8]
RVA/Human-wt/RWA/NGS-UFS-MRC-DPRU229/2012/G9P[8]
RVA/Human-wt/RWA/UFS-NGS-MRC-DPRU210/2012/G9P[8]
RVA/Human-wt/RWA/UFS-NGS-MRC-DPRU433/2013/G9P[8]
RVA/Human-wt/NPL/06N0397/2006/G12P[6]
RVA/Human-wt/NPL/5N0109/2005/G12P[6]
RVA/Human-wt/BEL/BE00029/2008/G1P[8]
RVA/Human-wt/AUS/CK00083/2008/G1P[8]
RVA/Human-wt/MOZ/HJM1646/2017/G1P[8]
RVA/Human-wt/ITA/PA93/12/2012/G12P[8]
RVA/Human-wt/ESP/SS257451/2012/G12P[8]
RVA/Human-wt/MWI/MW2-1254/2005/G1P[8]
RVA/Human-wt/JPN/Tokyo17-21/2017/G3P[8]
RVA/Human-wt/RWA/UFS-NGS-MRC-DPRU8058/2015/G9P[8]
RVA/Human-wt/RWA/UFS-NGS-MRC-DPRU8039/2015/G9P[8]
RVA/Human-wt/RWA/UFS-NGS-MRC-DPRU10026/2015/G9P[8]
RVA/Human-wt/RWA/UFS-NGS-MRC-DPRU213/2012/G9P[8]
RVA/Human-wt/RWA/UFS-NGS-MRC-DPRU541/2012/G9P[8]
RVA/Human-wt/RWA/UFS-NGS-MRC-DPRU413/2012/G9P[8]
RVA/Human-wt/RWA/UFS-NGS-MRC-DPRU425/2012/G9P[8]
RVA/Human-wt/RWA/UFS-NGS-MRC-DPRU238/2012/G9P[8]
RVA/Human-wt/RWA/UFS-NGS-MRC-DPRU543/2013/G9P[8]
Outgroup
0,001

VP3

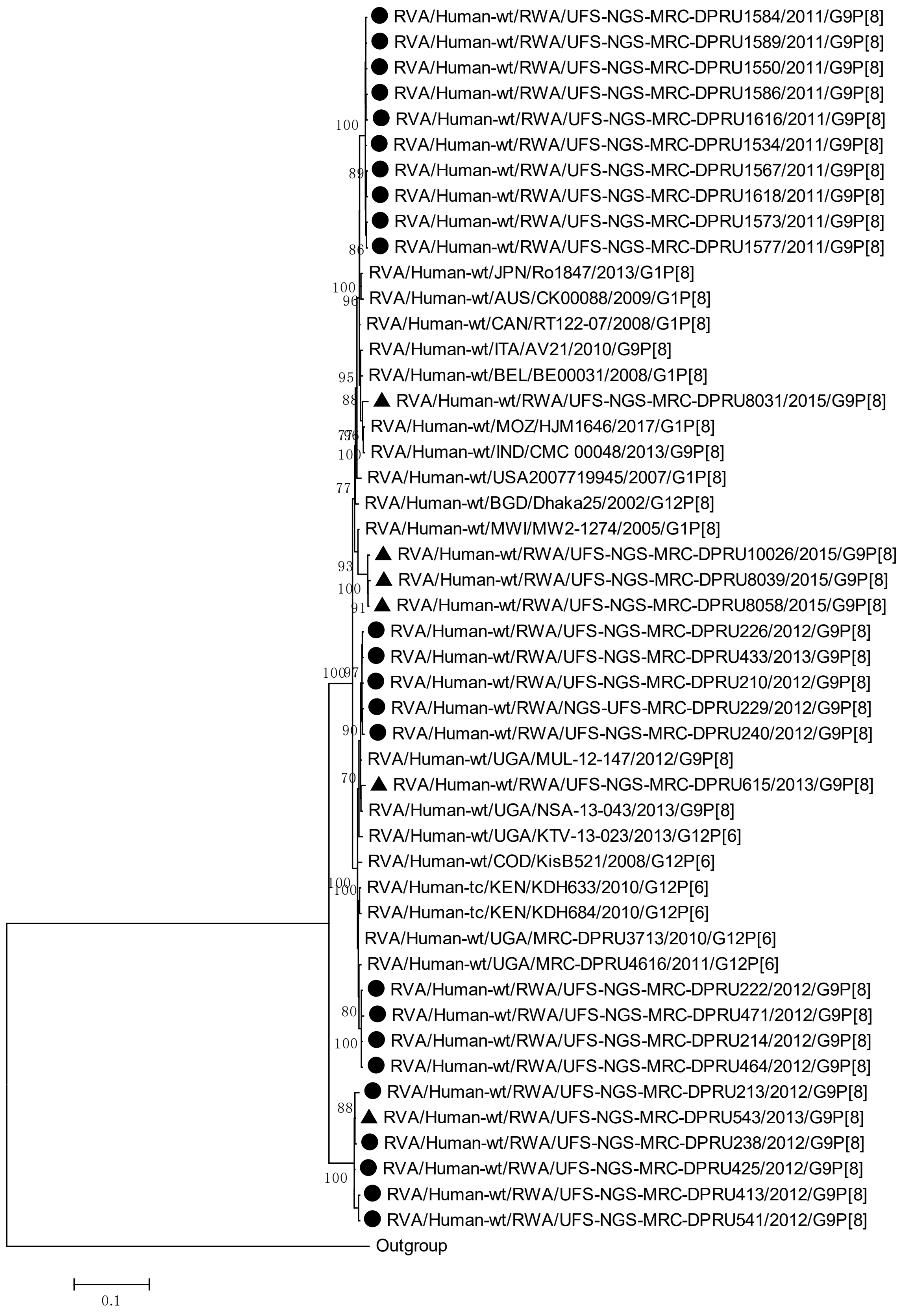
RVA/Human-wt/RWA/UFS-NGS-MRC-DPRU1584/2011/G9P[8]
RVA/Human-wt/RWA/UFS-NGS-MRC-DPRU1589/2011/G9P[8]
RVA/Human-wt/RWA/UFS-NGS-MRC-DPRU1550/2011/G9P[8]
RVA/Human-wt/RWA/UFS-NGS-MRC-DPRU1586/2011/G9P[8]
RVA/Human-wt/RWA/UFS-NGS-MRC-DPRU1616/2011/G9P[8]
RVA/Human-wt/RWA/UFS-NGS-MRC-DPRU1534/2011/G9P[8]
RVA/Human-wt/RWA/UFS-NGS-MRC-DPRU1567/2011/G9P[8]
RVA/Human-wt/RWA/UFS-NGS-MRC-DPRU1618/2011/G9P[8]
RVA/Human-wt/RWA/UFS-NGS-MRC-DPRU1573/2011/G9P[8]
RVA/Human-wt/RWA/UFS-NGS-MRC-DPRU1577/2011/G9P[8]
RVA/Human-wt/JPN/Ro1847/2013/G1P[8]
RVA/Human-wt/AUS/CK00088/2009/G1P[8]
RVA/Human-wt/CAN/RT122-07/2008/G1P[8]
RVA/Human-wt/ITA/AV21/2010/G9P[8]
RVA/Human-wt/BEL/BE00031/2008/G1P[8]
RVA/Human-wt/RWA/UFS-NGS-MRC-DPRU8031/2015/G9P[8]
RVA/Human-wt/MOZ/HJM1646/2017/G1P[8]
RVA/Human-wt/IND/CMC 00048/2013/G9P[8]
RVA/Human-wt/USA2007719945/2007/G1P[8]
RVA/Human-wt/BGD/Dhaka25/2002/G12P[8]
RVA/Human-wt/MWI/MW2-1274/2005/G1P[8]
RVA/Human-wt/RWA/UFS-NGS-MRC-DPRU10026/2015/G9P[8]
RVA/Human-wt/RWA/UFS-NGS-MRC-DPRU8039/2015/G9P[8]
RVA/Human-wt/RWA/UFS-NGS-MRC-DPRU8058/2015/G9P[8]
RVA/Human-wt/RWA/UFS-NGS-MRC-DPRU226/2012/G9P[8]
RVA/Human-wt/RWA/UFS-NGS-MRC-DPRU433/2013/G9P[8]
RVA/Human-wt/RWA/UFS-NGS-MRC-DPRU210/2012/G9P[8]
RVA/Human-wt/RWA/NGS-UFS-MRC-DPRU229/2012/G9P[8]
RVA/Human-wt/RWA/UFS-NGS-MRC-DPRU240/2012/G9P[8]
RVA/Human-wt/UGA/MUL-12-147/2012/G9P[8]
RVA/Human-wt/RWA/UFS-NGS-MRC-DPRU615/2013/G9P[8]
RVA/Human-wt/UGA/NSA-13-043/2013/G9P[8]
RVA/Human-wt/UGA/KTV-13-023/2013/G12P[6]
RVA/Human-wt/COD/KisB521/2008/G12P[6]
RVA/Human-tc/KEN/KDH633/2010/G12P[6]
RVA/Human-tc/KEN/KDH684/2010/G12P[6]
RVA/Human-wt/UGA/MRC-DPRU3713/2010/G12P[6]
RVA/Human-wt/UGA/MRC-DPRU4616/2011/G12P[6]
RVA/Human-wt/RWA/UFS-NGS-MRC-DPRU222/2012/G9P[8]
RVA/Human-wt/RWA/UFS-NGS-MRC-DPRU471/2012/G9P[8]
RVA/Human-wt/RWA/UFS-NGS-MRC-DPRU214/2012/G9P[8]
RVA/Human-wt/RWA/UFS-NGS-MRC-DPRU464/2012/G9P[8]
RVA/Human-wt/RWA/UFS-NGS-MRC-DPRU213/2012/G9P[8]
RVA/Human-wt/RWA/UFS-NGS-MRC-DPRU543/2013/G9P[8]
RVA/Human-wt/RWA/UFS-NGS-MRC-DPRU238/2012/G9P[8]
RVA/Human-wt/RWA/UFS-NGS-MRC-DPRU425/2012/G9P[8]
RVA/Human-wt/RWA/UFS-NGS-MRC-DPRU413/2012/G9P[8]
RVA/Human-wt/RWA/UFS-NGS-MRC-DPRU541/2012/G9P[8]
Outgroup
0.1

VP6

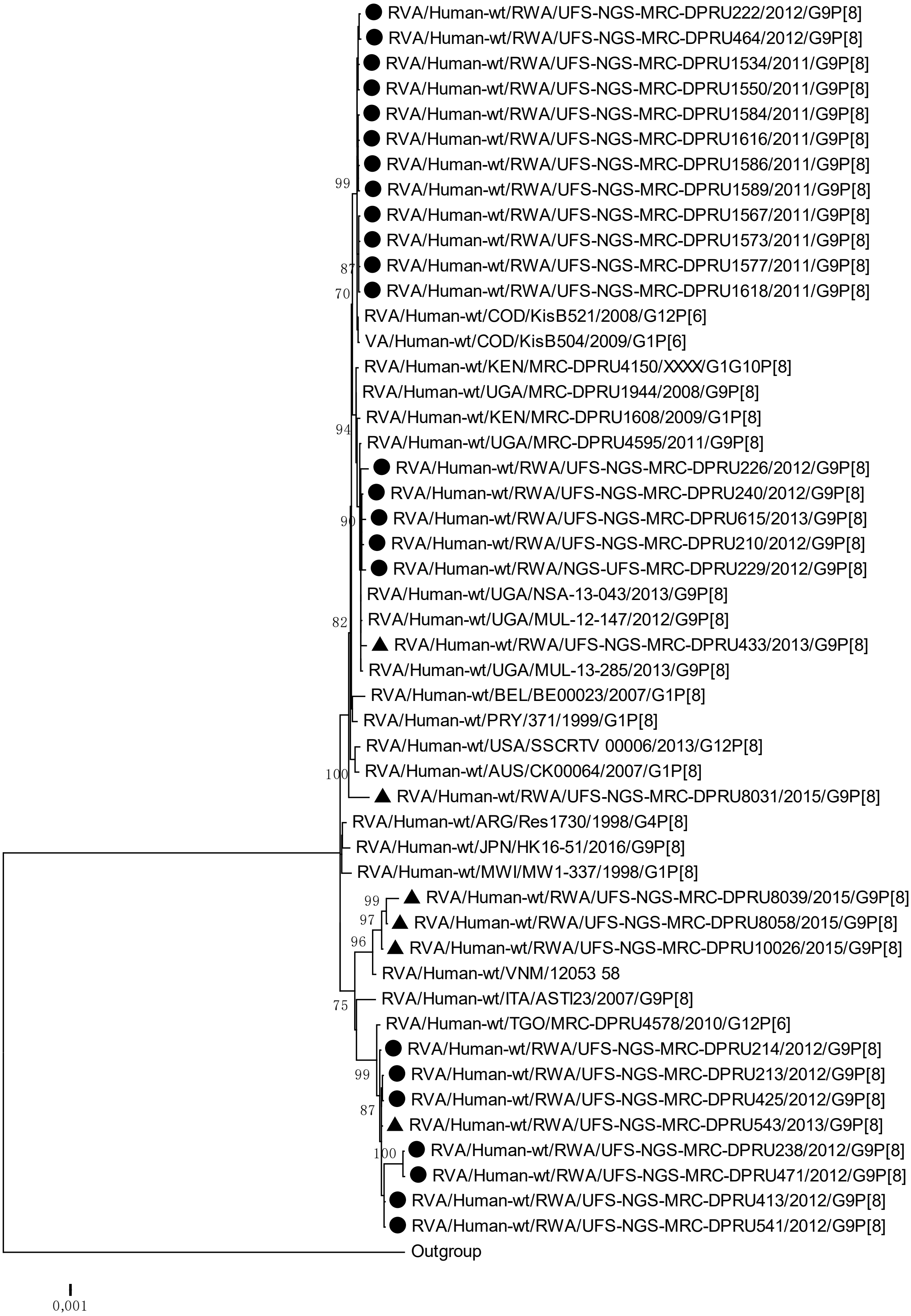
RVA/Human-wt/RWA/UFS-NGS-MRC-DPRU222/2012/G9P[8]
RVA/Human-wt/RWA/UFS-NGS-MRC-DPRU464/2012/G9P[8]
RVA/Human-wt/RWA/UFS-NGS-MRC-DPRU1534/2011/G9P[8]
RVA/Human-wt/RWA/UFS-NGS-MRC-DPRU1550/2011/G9P[8]
RVA/Human-wt/RWA/UFS-NGS-MRC-DPRU1584/2011/G9P[8]
RVA/Human-wt/RWA/UFS-NGS-MRC-DPRU1616/2011/G9P[8]
RVA/Human-wt/RWA/UFS-NGS-MRC-DPRU1586/2011/G9P[8]
RVA/Human-wt/RWA/UFS-NGS-MRC-DPRU1589/2011/G9P[8]
RVA/Human-wt/RWA/UFS-NGS-MRC-DPRU1567/2011/G9P[8]
RVA/Human-wt/RWA/UFS-NGS-MRC-DPRU1573/2011/G9P[8]
RVA/Human-wt/RWA/UFS-NGS-MRC-DPRU1577/2011/G9P[8]
RVA/Human-wt/RWA/UFS-NGS-MRC-DPRU1618/2011/G9P[8]
RVA/Human-wt/COD/KisB521/2008/G12P[6]
VA/Human-wt/COD/KisB504/2009/G1P[6]
RVA/Human-wt/KEN/MRC-DPRU4150/XXXX/G1G10P[8]
RVA/Human-wt/UGA/MRC-DPRU1944/2008/G9P[8]
RVA/Human-wt/KEN/MRC-DPRU1608/2009/G1P[8]
RVA/Human-wt/UGA/MRC-DPRU4595/2011/G9P[8]
RVA/Human-wt/RWA/UFS-NGS-MRC-DPRU226/2012/G9P[8]
RVA/Human-wt/RWA/UFS-NGS-MRC-DPRU240/2012/G9P[8]
RVA/Human-wt/RWA/UFS-NGS-MRC-DPRU615/2013/G9P[8]
RVA/Human-wt/RWA/UFS-NGS-MRC-DPRU210/2012/G9P[8]
RVA/Human-wt/RWA/NGS-UFS-MRC-DPRU229/2012/G9P[8]
RVA/Human-wt/UGA/NSA-13-043/2013/G9P[8]
RVA/Human-wt/UGA/MUL-12-147/2012/G9P[8]
RVA/Human-wt/RWA/UFS-NGS-MRC-DPRU433/2013/G9P[8]
RVA/Human-wt/UGA/MUL-13-285/2013/G9P[8]
RVA/Human-wt/BEL/BE00023/2007/G1P[8]
RVA/Human-wt/PRY/371/1999/G1P[8]
RVA/Human-wt/USA/SSCRTV 00006/2013/G12P[8]
RVA/Human-wt/AUS/CK00064/2007/G1P[8]
RVA/Human-wt/RWA/UFS-NGS-MRC-DPRU8031/2015/G9P[8]
RVA/Human-wt/ARG/Res1730/1998/G4P[8]
RVA/Human-wt/JPN/HK16-51/2016/G9P[8]
RVA/Human-wt/MWI/MW1-337/1998/G1P[8]
RVA/Human-wt/RWA/UFS-NGS-MRC-DPRU8039/2015/G9P[8]
RVA/Human-wt/RWA/UFS-NGS-MRC-DPRU8058/2015/G9P[8]
RVA/Human-wt/RWA/UFS-NGS-MRC-DPRU10026/2015/G9P[8]
RVA/Human-wt/VNM/12053 58
RVA/Human-wt/ITA/ASTI23/2007/G9P[8]
RVA/Human-wt/TGO/MRC-DPRU4578/2010/G12P[6]
RVA/Human-wt/RWA/UFS-NGS-MRC-DPRU214/2012/G9P[8]
RVA/Human-wt/RWA/UFS-NGS-MRC-DPRU213/2012/G9P[8]
RVA/Human-wt/RWA/UFS-NGS-MRC-DPRU425/2012/G9P[8]
RVA/Human-wt/RWA/UFS-NGS-MRC-DPRU543/2013/G9P[8]
RVA/Human-wt/RWA/UFS-NGS-MRC-DPRU238/2012/G9P[8]
RVA/Human-wt/RWA/UFS-NGS-MRC-DPRU471/2012/G9P[8]
RVA/Human-wt/RWA/UFS-NGS-MRC-DPRU413/2012/G9P[8]
RVA/Human-wt/RWA/UFS-NGS-MRC-DPRU541/2012/G9P[8]
Outgroup
99
87
70
94
90
82
100
99
97
96
75
99
87
100
0,001
